# Local administration of liposomal-based Srpx2 gene therapy reverses pulmonary fibrosis by blockading fibroblast-to-myofibroblast transition

**DOI:** 10.7150/thno.61085

**Published:** 2021-05-13

**Authors:** Qi Wang, Juan Liu, Yinan Hu, Ting Pan, Yongjian Xu, Jun Yu, Weining Xiong, Qing Zhou, Yi Wang

**Affiliations:** 1 Department of Respiratory and Critical Care Medicine, Key Laboratory of Pulmonary Diseases of National Health Commission, Key Site of National Clinical Research Center for Respiratory Disease, Wuhan Clinical Medical Research Center for Chronic Airway Diseases, Tongji Hospital, Tongji Medical College, Huazhong University of Sciences and Technology, 1095 Jiefang Ave, Wuhan 430030, China.; 2 Department of Pulmonary and Critical Care Medicine, Center of Respiratory Medicine, China-Japan Friendship Hospital, 100029, Beijing, China.; 3 Department of Thoracic Surgery, Tongji Hospital, Tongji Medical College, Huazhong University of Sciences and Technology, 1095 Jiefang Ave, Wuhan 430030, China.; 4 Department of Respiratory and Critical Care Medicine, Shanghai Key Laboratory of Tissue Engineering, Shanghai Ninth People's Hospital, Shanghai Jiaotong University School of Medicine, 639 Zhizaoju Lu, Shanghai, 200011, China.; 5 The Center for Biomedical Research, Tongji Hospital, Tongji Medical College, Huazhong University of Sciences and Technology, 1095 Jiefang Ave, Wuhan 430030, China.

**Keywords:** idiopathic pulmonary fibrosis, fibroblasts, myofibroblasts, SRPX2, liposomes

## Abstract

Idiopathic pulmonary fibrosis (IPF) is a chronic and progressive fatal interstitial lung disease characterized by abnormal transition and proliferation of fibroblasts. The uncontrolled transition of fibroblasts, commonly known as myofibroblasts, are the principal source of the enormous extracellular matrix (ECM) depositing in lung parenchyma, leading to gradual failure of gas exchange and mortality of the patients. However, up to now, rare effective therapeutic strategies have been developed to blockade fibroblast-to-myofibroblast transition (FMT) in IPF.

**Method:** We illustrated that the lungs originated from IPF patients and mice with pulmonary fibrosis are characterized by the overexpression of sushi-repeat-containing protein, X-linked 2 (SRPX2). Further functionality studies identified the pivotal role of SRPX2 in FMT. Mechanistically, SRPX2 was involved in a TGFβR1/SMAD3/SRPX2/AP1/SMAD7 positive feedback loop. Specifically, SRPX2 was upregulated by TGF-β1 in a TGFβR1/SMAD3-dependent manner, after which SRPX2 in turn repressed the expression of AP1, subsequently minimized SMAD7 expression, through which it reduced the formation of inhibitory complex with TGFβR1 and enhanced SMAD signaling pathway to promote FMT and exacerbate pulmonary fibrosis. Notably, intratracheal administration of siRNA-loaded liposomes could effectively suppress the expression of Srpx2 in the lung and remarkably protect mice against BLM-induced pulmonary fibrosis, concomitant with a significant reduction of FMT.

**Results:** Accordingly, these data indicate that Srpx2 plays an essential role in the pathogenesis of pulmonary fibrosis and suggests the strategy aiming at silencing Srpx2 could be a promising therapeutic approach against pulmonary fibrosis in clinical settings.

## Introduction

Idiopathic pulmonary fibrosis (IPF), a fatal lung interstitial disease, is characterized by progressive scarring of the pulmonary parenchyma with a median survival of only 2-3 years after diagnosis 1. Although the new antifibrotic agents of Ofev (nintedanib) and Esbriet (pirfenidone) approved by FDA improve the wellbeing of patients, the prognosis of IPF remains poor with 5-year mortality rates still ranging from 70 to 80%^2^. Therefore, it is urgent to develop effective therapeutic approaches for IPF in clinical settings.

Current pathogenic theories demonstrate that fibroblast-to-myofibroblast transition (FMT) plays a central role in the pathogenesis of IPF, featured by uncontrolled production of extracellular matrix (ECM) depositing in lung parenchyma 3, 4. With lung injury, fibroblasts are exposed to pro-fibrotic factors such as transforming growth factor-beta 1 (TGF-β1), connective tissue growth factor (CTGF) and platelet derived growth factor (PDGF), and then wildly differentiate to myofibroblasts in abundance, leading to enormous deposition of ECM in the interstitium of lung, which thereby results in gradual failure of gas exchange and mortality of the patients 5. Among above mentioned pro-fibrotic factors, TGF-β is the most potent mediator for FMT in the fibrogenic process 6, 7. TGF-β binds to TGF-β receptor 2 (TGFβR2) forming a complex. And then, TGFβR1 is recruited into the complex and activated by TGFβR2. Subsequently, the intracellular signaling pathway of TGF-β receptors is mediated by canonical pathway (Smad proteins) or non-canonical pathways (MAPK and Rho family members) 8. Both canonical and non-canonical pathways contribute to the differentiation of fibroblasts into alpha-smooth muscle actin (α-SMA) positive myofibroblasts and production of ECM 9, 10. Even though suppression of TGF-β is considered theoretically as a feasible approach for the treatment of pulmonary fibrosis, the side effects are unacceptably serious. As a result, no such agents have been applied in clinical settings so far 9.

To identify more promising targets for the blockade of FMT induced by TGF-β1, herein, we employed deep RNA-Sequence to delineate the transcriptome changes in myofibroblasts. Notably, sushi repeat-containing protein X-linked 2 (SRPX2), a chondroitin sulfate proteoglycan, was strikingly overexpressed in TGF-β1-induced fibroblasts. Although recent studies have revealed the critical role of SRPX2 in the occurrence and progression of various cancers 11, 12, the functionality of SRPX2 on FMT remains unknown. Based on sequencing results, we hypothesized that SRPX2 might impact the process of FMT and could be a potential target for the treatment of IPF.

Previous studies 13, 14, including ours 15, demonstrated that cationic liposomes are promising strategies for the siRNA-based therapy of various disease. Especially for pulmonary fibrosis, we have verified that liposomes administrated by intratracheal injection, equivalent to clinical atomization treatment, preferred to accumulate in the fibrotic lesion, in which consists of abundant fibroblasts and/or myofibroblasts. Therefore, in the current study, we attempted to employ *Srpx2* siRNA-loaded liposomes to suppress FMT for the treatment of pulmonary fibrosis. Indeed, both of *in vitro* and *in vivo* studies provided compelling evidence that suppression of *Srpx2* by siRNA-loaded liposomes significantly protected mice against BLM-induced lung injury and fibrosis, coupled with the pronounced reduction of fibroblast transition in the lung. In a mechanistic study, for the first time, we demonstrated that silencing SRPX2 expression could repress fibroblast differentiation by blocking the AP1/SMAD7/SMAD2/3 signaling axis. Collectively, our data support the notion that Srpx2 plays a critical role in the progression of pulmonary fibrosis and the liposomes-based strategy aiming at silencing Srpx2 could be a viable therapeutic approach against pulmonary fibrosis in clinical settings.

## Methods

### Materials

Antibodies against Collagen type I, α-SMA, SMAD2, SMAD3 were purchased from Cell Signaling Technology (MA, USA). Antibodies against p-SMAD2, p-SMAD3 and p-SMAD2/3 were obtained from Boster Biological Technology Co., ltd (Wuhan, China). Antibodies against SRPX2, β-ACTIN, SMAD7 and FIBRONECTIN were acquired from Proteintech Group, Inc (IL, USA). Cholesterol and DSPC were acquired from Sigma-Aldrich, Inc. (St. Louis, MO) and mPEG2000-DMG (MW2660) was purchased from NOF Co., Ltd. (Kawasaki Japan). The cationic lipidoid C12-200 was generated through ring opening of epoxides by amine substrates with a previously reported method 16. The selective inhibitor of p-SMAD3 (SIS3-HCL), the selective inhibitor of TGF-β Receptor type I receptor (SB-431542) and Recombinant Human TGF-β1 were obtained from MedChemExpress (NJ, USA). T-5224, a selective inhibitor targeting AP-1, was obtained from Selleck Chemicals (Texas, USA). BLM was obtained from Hisun Pharmaceutical Co., Ltd (Zhejiang, China). RT-PCR assay kit was supplied by Takara (Liaoning, China). A hydroxyproline assay kit was obtained from BioVision (CA, USA).

### Human samples

Lung tissues were collected from patients with non-small cell lung cancer (NSCLC, n = 6, incisal edge > 5cm) and patients with IPF (n = 6) in Tongji Hospital after receiving informed consent. IPF was diagnosed according to the American Thoracic Society (ATS)/European Respiratory Society (ERS) consensus diagnostic criteria 17. The experiments were approved by the Human Assurance Committee of Tongji Hospital. Clinical data and the results of pulmonary function tests are provided in Table 1.

### RNA interference

Three siRNAs targeting *SRPX2* (named si-*SRPX2*_001, si-*SRPX2*_002, and si-*SRPX2*_003, respectively) and a nontargeting control siRNA (named scramble siRNA) were purchased from RiboBio (Guangdong, China). The specific *SRPX2*-targeted siRNA sequences were as follows: si-*SRPX2*_001: 5'-CAG ATG AAA GCT ACA ATG A-3', si-*SRPX2*_002: 5'-CAG ATG AAA GCT ACA ATG A-3', and si-*SRPX2*_3: 5'-GAG GAA ATC TTC ACA TTC A-3'. For transfection, Lipofectamine 3000 (Invitrogen, CA, USA) was used according to previously reported 18.

### Preparation and characterization of *Srpx2* siRNA-loaded liposomes

Liposomes were raised as carriers to encapsulate siRNA. The siRNA was dissolved in citrate buffer (10 mM, pH = 3) and rapidly mixed with a lipid mixture by vortexing. The lipid mixture was composed of C12-200, cholesterol, DSPC and mPEG-DMG dissolved in ethanol at a molar ratio of 50:38.5:10:1.5. The unentrapped siRNA was removed by ultrafiltration centrifugation. The entrapment efficiency was measured by RiboGreen assay. SiRNA-loaded liposomes were used after being diluted with PBS. The characteristics of liposomes (hydrodynamic diameter, zeta potential, morphology and stability) were listed in Supplemental Figure 3. These features were measured by dynamic light scattering (DLS) (Malvern Zetasizer Nano-ZS, UK). The morphology of the liposomes was investigated by transmission electron microscopy (TEM, Tecnai G2-20).

### BLM-mediated induction of pulmonary fibrosis

All male C57BL/6 mice (8-10 weeks old) were purchased from Beijing Vital River Laboratory Animal Technology Co., Ltd. (Beijing, China). The mice were all housed in a specific pathogen-free (SPF) animal facility at Tongji Hospital in a 12:12 h light/dark photocycle with sterile acidified water and irradiated food. All animal experimental procedures were allowed by the Animal Care and Use Committee at Tongji Hospital. The mice were anesthetized with 1% pentobarbital sodium (60 mg/kg) by intraperitoneal injection, and the 2.5 mg/kg BLM diluted in PBS or PBS alone was administered via intratracheal injection as previously reported^19^. On days 14 and 18 following BLM induction, *Srpx2* siRNA-loaded liposomes, scramble siRNA-loaded liposomes or unloaded liposomes (dosage of siRNA, 1 mg/kg) were injected via intratracheal to the corresponding group of the mice. Eventually, all mice were euthanized 21 days after BLM injection for analysis of pulmonary fibrosis.

### Histopathological assessment

The left lungs were collected and fixed in 4% neutral buffered paraformaldehyde for 24h at room temperature, followed by embedded in paraffin. Subsequently, the lungs were sliced into 5 µm sections and stained with hematoxylin and eosin (H&E), Sirius red and Masson's trichrome as previously reported 20. Ashcroft scale was used to score the severity of pulmonary fibrosis on a scale from 0 to 8 in a blinded fashion by two pathologists.

### Quantitative analysis of hydroxyproline

A hydroxyproline assay kit (BioVision, CA, USA) was used to measure the hydroxyproline content of the lungs to assess the lung collagen deposition according to previously reported 21.

### Primary lung fibroblast cell culture

Human lung tissues from IPF patients and control subjects were minced and allowed to adhere to the bottom of the culture flask. Samples were cultured for 24h after attachment to the culture flask bottom, in Dulbecco's Modified Eagle's Medium (DMEM) supplemented with 10% (v/v) fetal bovine serum (FBS; Gibco, USA) at 37 °C in a 5% CO_2_ humidified incubator. The cell culture medium was refreshed every 3 days, and the cells were passaged at a 1:2 ratio every 3-4 days.

### *In vivo* biodistribution of the liposomes

DiR-labelled liposomes were constructed as the above method. And then, the prepared liposomes were intratracheal injected to the C57/B6 mice. The mice were then anesthetized and imaged at different time points (0 h, 2 h, 8 h, 3 d, 6 d) by an *in vivo* imaging system (IVIS Lumina XR, SI Imaging, AZ, USA) (excitation: 745 nm, emission: 830 nm) as previous reported 15. After 6 d, the mice were sacrificed, and the organs and serum were harvested for *ex vivo* fluorescence imaging and biochemical detection, respectively.

### RNA deep sequencing (RNA-seq)

RNAs from mouse lung fibroblasts after TGF-β1 stimulated for 24 h were extracted using an RNA isolation kit (QIAGEN, Shanghai, China). RNA quality and integrity were determined using the Nanodrop 2000 Spectrophotometer (Thermo Scientific, MA, USA) and Agilent Bioanalyser (DNA TECH, CA, USA). RNA-seq libraries were multiplexed and loaded per lane into the Illumina HiSeq flow cell v3. All sequencing protocols were carried out as the manufacturer's instructions using the Illumina HiSeq 1000 and HiSeq control software.

### Quantitative Real-time PCR

Total RNA was extracted from fibroblasts and the mice lungs with TRlzol reagent (Takara, Dalian, China) as previously reported 22. A NanoDrop 2000 spectrophotometer (Thermo Scientific, MA, USA) was used to measure the RNA quantity and quality. Complementary DNA synthesis was performed using a M-MLV reverse transcriptase kit (Invitrogen, CA, USA). Real-time PCR was performed on a CFX96 Real-time PCR detection system (Bio-Rad, CA, USA) using SYBR Green Mix (Takara, Dalian, China). The following primer sequences were used: human *SRPX2* forward, 5′- TGG CTG GTT GAT TTT GTA GAG AAA-3′ and reverse, 5′- TAG AAA AGA GTT AGG TGT CAC ATT GAA TAA-3′; human *FIBRONECTIN* forward, 5′- GAT GTC CGA ACA GCT ATT TAC CA-3′ and reverse, 5′- CGA CCA CAT AGG AAG TCC CAG-3′; human *COL1A1* forward, 5′- GAG GGC CAA GAC GAA GAC ATC-3′ and reverse, 5′- CAG ATC ACG TCA TCG CAC AAC-3′; human *ACTA2* forward, 5′-GAC GCT GAA GTA TCC GAT AGA ACA CG-3′ and reverse, 5′- CAC CAT CTC CAG AGT CCA GCA CAA T-3′; human *ACTB* forward, 5′-AGC GAG CAT CCC CCA AAG TT-3′ and reverse, 5′-GGG CAC GAA GGC TCA TCA TT-3′; human *AP-1* forward, 5′- CAA ACC TCA GCA ACT TCA ACC-3′ and reverse, 5′- CTG GGA CTC CAT GTC GAT G-3′; human *SMAD7* forward, 5′- ATG TTC AGG ACC AAA CGA TCT-3′ and reverse, 5′- GGA TGG TGG TGA CCT TTG G-3′.mouse *Srpx2* forward, 5′-ATG AAG TGT TCC AGC GAT GGT GA-3′ and reverse, 5′-TGG CAT ACT CGG GCA GGA CTA C-3′; mouse *Fibronectin* forward, 5′- GAT GTC CGA ACA GCT ATT TAC CA-3′ and reverse, 5′-CCT TGC GAC TTC AGC CAC T-3′; mouse *Col1a1* forward, 5′-TAA GGG TCC CCA ATG GTG AGA-3′ and reverse, 5′-GGG TCC CTC GAC TCC TAC AT-3′; mouse *Acta2* forward, 5′-GGA CGT ACA ACT GGT ATT GTG C-3′ and reverse, 5′- TCG GCA GTA GTC ACG AAG GA-3′; mouse *Actb* forward, 5′-GCC ACA GCA CTC CAT CGA C-3′ and reverse, 5′-GTC TCC GAT CTG GAA AAC GC-3′, mouse *AP-1* forward, 5′-AAG ATG GAA ACG ACC TTC TAC G-3′ and reverse, 5′-CTT AGG GTT ACT GTA GCC GTA G-3′; mouse *Smad7* forward, 5′-CTG TGT TGC TGT GAA TCT TAC G-3′ and reverse, 5′- GAG ACT CTA GTT CAC AGA GTC G-3′.

### Western blot analysis

Fibroblasts and lung tissues were homogenized in RIPA lysis buffer. Proteins were subjected to Western blot with the indicated primary antibodies using established techniques 23. The gray values were analyzed with ImageJ software.

### Immunofluorescence analysis

For the immunofluorescence experiment, the slides were treated with antibody at 4 °C overnight. The primary antibodies used for staining were as follows: mouse anti-SRPX2 (1:100), rabbit anti-FIBRONECTIN (1:100), rabbit anti-COLLAGEN I (1:100), rabbit anti-α-SMA (1:100), and rabbit anti-p-SMAD2/3 (1:100). All slides were incubated with an Alexa 488-conjugated anti-mouse and Alexa 594-labeled anti-rabbit antibody (Invitrogen, CA, USA, 1:400) after washed with PBS. And then, the nuclei were counterstained with DAPI (4'-6-diamidino-2-phenylindole) for 5 min. Finally, the slides were analyzed under a fluorescence microscope (Olympus, Shinjuku, Japan).

### Cell proliferation assay

Human pulmonary fibroblasts (HPFs) were purchased from ScienCell Research Laboratories, Inc (Nanjing, China) and cultured in 96-well plates at a density of 2×10^3^ cells/well. Cell proliferation was then measured using the EdU proliferation assay (Ribobio, Guangzhou, China) as previously reported 4. Briefly, 24 h after being seeded in the plates, cells were labeled with EdU for 2 h at 37 °C, treated with 100 µL of Apollo reaction cocktail and stained with 100 µL of Hoechst 33342. Finally, the cells were observed under a fluorescence microscope (Olympus, Shinjuku, Japan).

### Cell migration assay

Cell migration assays were performed using Transwell inserts with a membrane with a pore size of 8.0 mm (Corning, MA, USA) according to previously reported methods 24. The cells were resuspended in 2% serum-containing medium and seeded into the upper chambers at a density of 2.5×10^4^ cells/well. The lower chambers were filled with culture medium containing 10% FBS to function as a chemoattractant. After incubation at 37 °C for 24 h, the cells migrated through the membrane filter and were stained with 0.1% crystal violet (Sigma-Aldrich, St. Louis, MO, USA).

### Statistical analyses

Experimental data are expressed as the Mean ± SEM. All statistical analyses were performed using GraphPad Prism (San Diego, CA, USA). Two experimental groups were compared using the Student's t test or the Student's t test with Welch's correction for unpaired data. More than two groups were compared using one-way ANOVA with Bonferroni's correction. *p*<0.05 was used to indicate statistical significance.

## Results

### FMT is characterized by SRPX2 overexpression during the progression of IPF

**Figure 1:**
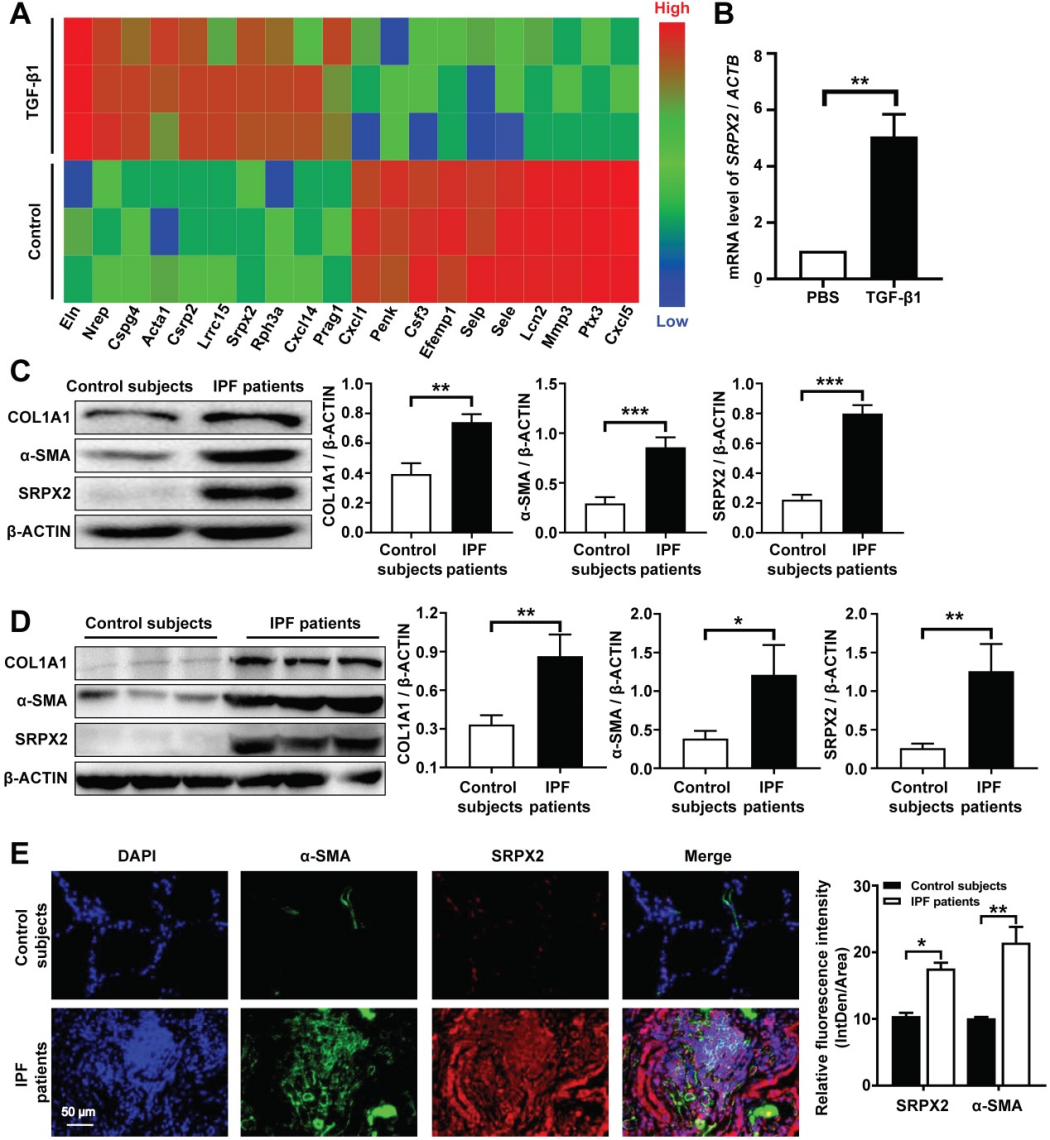
** Analysis of SRPX2 expression in patients with IPF. A**: A heat map for the differentially expressed genes identified by RNA-seq analysis during the course of FMT. The color of the heat map represents the fold enrichment in each sample. **B**: RT-PCR analysis of Srpx2 expression in fibroblasts following TGF-β1 induction. **C**: Western blot results for analysis of COL1A1, α-SMA and SRPX2 expression in fibroblasts derived from control subjects and IPF patients. **D**: Western blot analysis of COL1A1, α-SMA and SRPX2 expression in the lungs of control subjects and IPF patients. **E**: Representative results for co-immunostaining of SRPX2 and α-SMA in the lung sections from patients with IPF (n = 6) and control subjects (n = 6). The nuclei were stained blue by DAPI, and the images were taken under original magnification ×400. The data are represented as the mean ± SEM. *, p < 0.05; **, p < 0.01; ***, p < 0.001.

It is well-documented that FMT is pivotal for the pathogenesis of IPF 25. However, the underlying molecular mechanisms of FMT are still masked. To dissect this gap, a deep-RNA sequencing was conducted to delineate the transcriptome changes between fibroblasts and myofibroblasts. For sequencing, mouse lung fibroblasts (MLFs) were differentiated to myofibroblasts under the stimulation of TGF-β1 for 24 h. Interestingly, strikingly differential expression of Srpx2 family genes (Srpx2, Sele and Selp) were identified in top 20 genes significantly altered between fibroblasts and myofibroblasts (Figure 1A). Specifically, Srpx2 was overexpressed, while Sele and Selp were downregulated, indicating that Srpx2 family could be involved in the process of FMT. To confirm this notion, we next detected the expression of Srpx2 in MLFs by RT-PCR. In line with above observation, MLF treated with TGF-β1 exhibited a remarkably increased Srpx2 expression (Figure 1B). Notably, fibroblasts derived from IPF patients' lung also exhibited a higher level of SRPX2 than that from control subjects, along with markedly enhanced expressions of the fibrotic markers, COL1A1 and α-SMA (Figure 1C). Consistent with the *in vitro* results, SRPX2 was indeed up-regulated in IPF patients' lung compared to control subjects (Figure 1D). Additionally, the co-immunostaining results further reinforced the conclusion that SRPX2 (red) was highly expressed in α-SMA^+^ (green) myofibroblasts (Figure 1E). Taken together, all above data support the notion that SRPX2 was extremely overexpressed in fibroblasts originated from IPF patients' lung and might be involved in the process of FMT.

### Suppression of SRPX2 could inhibit FMT process in HPFs

**Figure 2:**
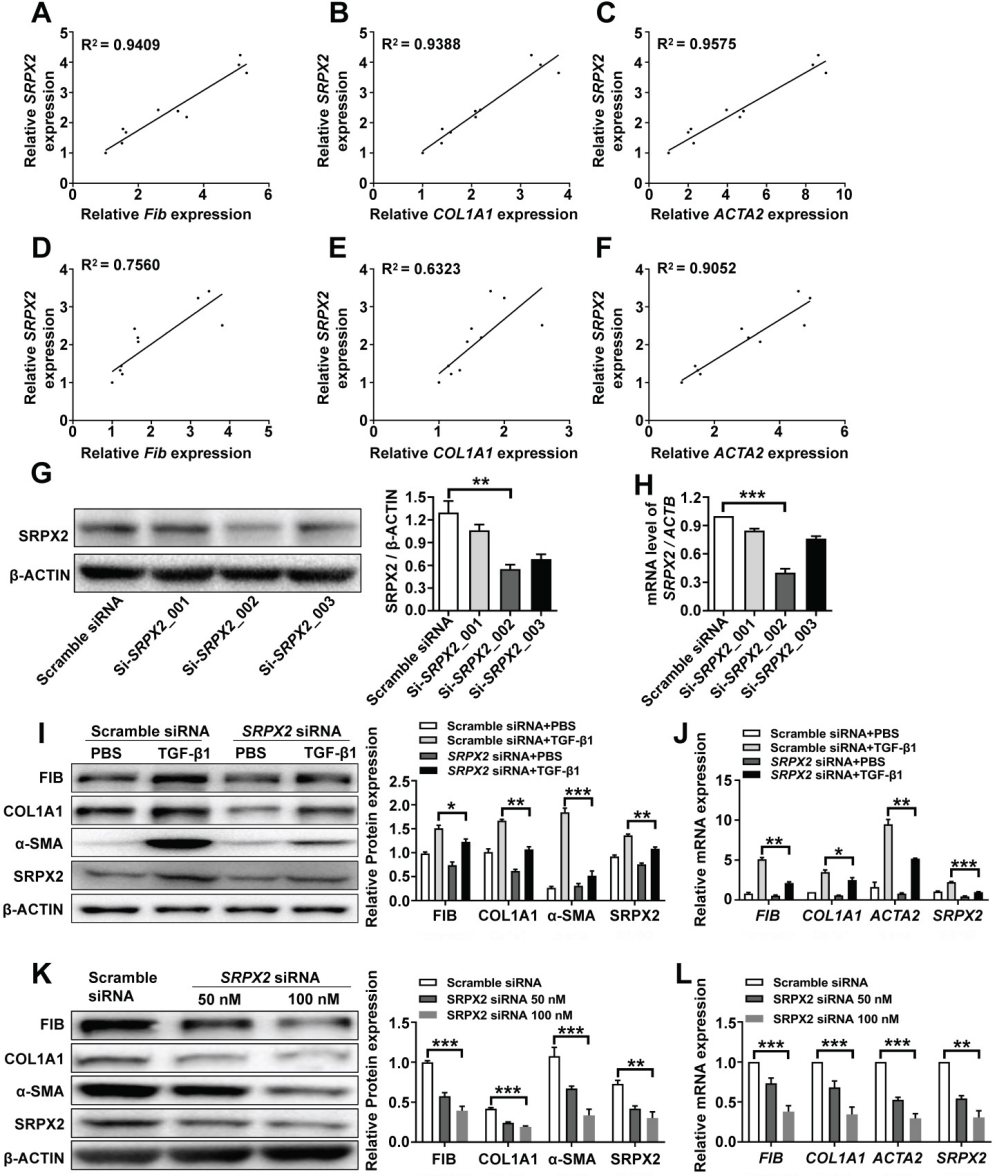
** Knockdown of SRPX2 attenuated FMT. A-C**: RT-PCR analysis of the correlation between *SRPX2* and *FIBRONECTIN* **(A)**, *COL1A1* **(B)** and *ACTA2* **(C)** expression following various doses of TGF-β1 induction for 24h. **D-F**: Results for time-course RT-PCR analysis of the correlation between *SRPX2* and *FIBRONECTIN* **(D)**, *COL1A1* **(E)** and *ACTA2* **(F)** expression after TGF-β1 stimulation (10 ng/ml). **G-H**: Western blot **(G)** and RT-PCR** (H)** analyses of the interference efficiency of *SRPX2* siRNAs in HPFs.** I-J**: Western blot** (I)** and RT-PCR** (J)** results for analysis of FIBRONECTIN, COL1A1 and α-SMA expression in *SRPX2* siRNA or Scrambled siRNA transduced HPFs following TGF-β1 induction. **K-L**: Western blot** (K)** and RT-PCR** (L)** analysis of FIBRONECTIN, COL1A1 and α-SMA expression in *SRPX2* siRNA or Scrambled siRNA treated fibroblasts originated from IPF patients. The data are represented as the mean ± SEM of three independent experiments. *, p < 0.05; **, p < 0.01; ***, p < 0.001.

Since fibroblasts from IPF patients were featured with an enhanced expression of SRPX2, we explored whether SRPX2 participate in regulating functionalities of fibroblasts. Firstly, the correlation between SRPX2 and FMT was revealed. After treating HPFs with various doses of TGF-β1, the expressions of SRPX2 and FMT related genes (*FIBRONECTIN*, *COL1A1* and *ACTA2*) were quantified by RT-PCR. Interestingly, the expression level of FMT related genes illustrated a positive correlation with the expression of SRPX2 (Figure 2A-C). Similar tendency was observed when the fibroblast was incubated with TGF-β1 for different time points (Figure 2D-F). Based on the above data, we speculated that SRPX2 could be a key regulation factor during the course of FMT. To verify this hypothesis, SRPX2 loss-of-function assays were conducted in HPFs. Si-*SRPX2*_002 was selected for the following study due to its optimal silencing efficacy (Figure 2G-H). After knocked down SRPX2 and treated with TGF-β1, HPFs were collected for Western blot and RT-PCR analyses. Remarkably, significantly lower expressions of FMT related proteins were demonstrated in SRPX2 siRNA transfected HPFs, indicating that SRPX2 could aggravate the process of FMT (Figure 2I-J). Furthermore, fibroblasts derived from IPF patients' lung were used to confirm the effects of *SRPX2* siRNA. In line with the results obtained from HPFs, silence of SRPX2 reversed the FMT process in a dose-depend manner (Figure 2K-L). Importantly, the immunostaining analysis further showed the similar results (Supplementary Figure 1). Additionally, we also assessed the impacts of SRPX2 on the proliferation and migration of HPFs by EdU staining and Transwell assay, respectively. Notably, loss of SRPX2 significantly suppressed the proliferation and migration of HPFs (Supplementary Figure 2A and B). Collectively, suppression of SRPX2 in fibroblasts could not only abrogate the TGF-β1-induced FMT, but also the proliferation and migration of HPFs.

### The overexpression of SRPX2 was induced by TGF-β1 in a TGFβRI/SMAD3-dependent manner

**Figure 3:**
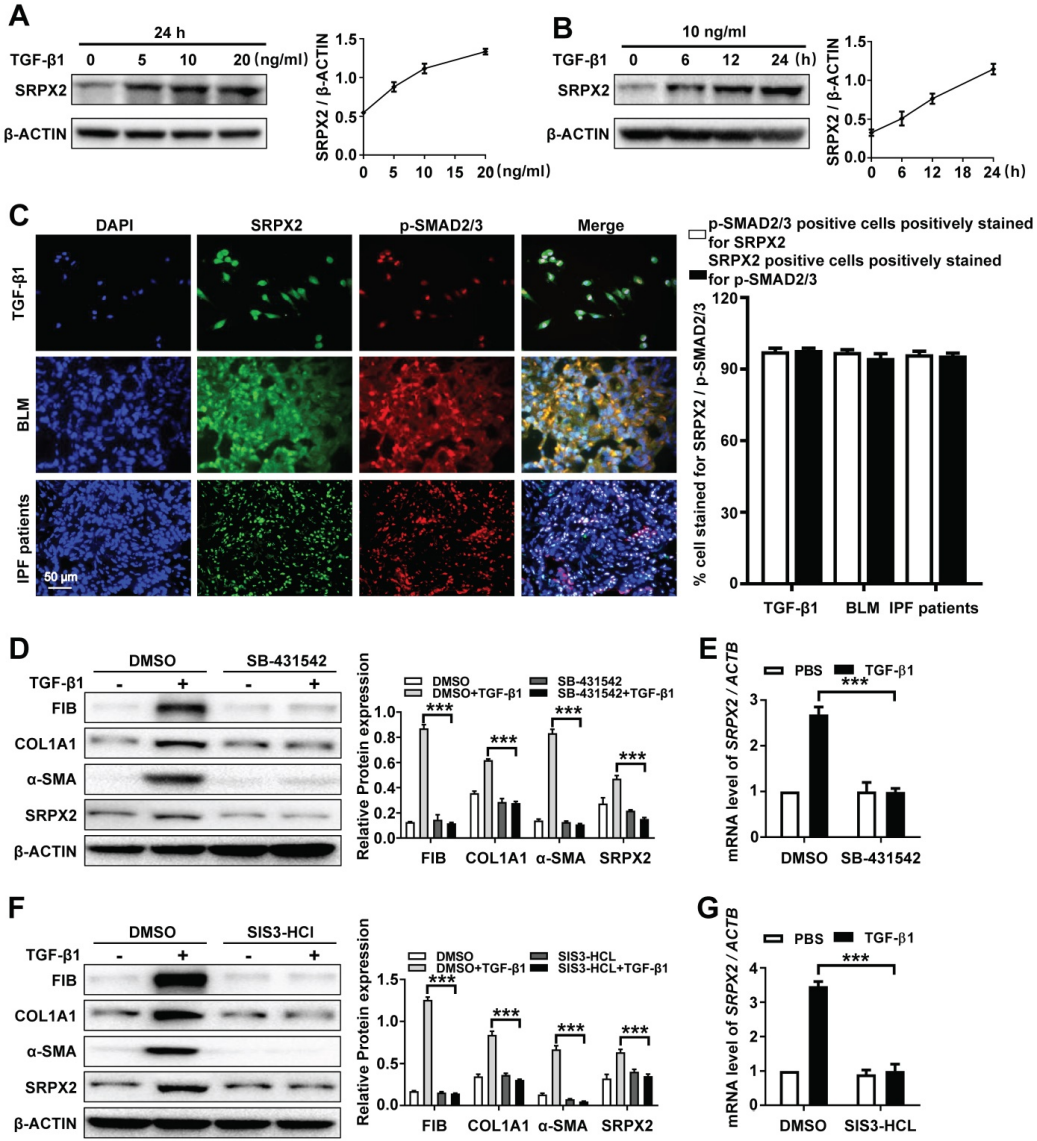
** SRPX2 is elevated in fibroblasts in a TGF-β/SMADs manner. A**: Western blot analysis of SRPX2 expression in HPFs following different dose of TGF-β1 induction for 24 h. **B**: Results for time-course Western blot analysis of SRPX2 expression in HPFs following TGF-β1 (10 ng/ml). **C**: Results for co-immunostaining of SRPX2 and p-SMAD2/3 in HPFs following TGF-β1 induction for 1h (up), lung sections of pulmonary fibrosis mice (middle), and lung sections from IPF patients (down). The nuclei were stained blue by DAPI, and the images were taken under original magnification ×400. **D-E**: Western blot** (D)** and RT-PCR** (E)** analysis of SRPX2 expression in HPFs pre-treated with SB431542 treatment following TGF-β1 induction. **F-G**: Western blot** (F)** and RT-PCR** (G)** analysis of SRPX2 expression in HPFs pre-treated with SIS3-HCL following TGF-β1 induction. The data are represented as the mean ± SEM of three independent experiments. ***, p < 0.001.

Based on the above observations, we attempted to explore the underlying mechanism of the up-regulated SRPX2 in FMT. As the sequencing result showed (Figure 1A), the expression of SRPX2 could be induced by TGF-β1. To further identify the correlation between SRPX2 and TGF-β1, the HPFs were treated with various concentration of TGF-β1 for different time points. Consistently, TGF-β1 induced SRPX2 overexpression in a dose and time-dependent manner, which suggested that TGF-β signaling pathway might contribute to the overexpression of SRPX2 during FMT (Figure 3A and B). Therefore, co-immunostaining of SRPX2 and p-SMAD2/3, key factors in TGF-β signaling pathway, was next conducted in TGF-β1 treated HPFs, BLM induced mice's lung sections and IPF patients' lung sections, respectively. Indeed, it is obviously noted that SRPX2 (green) colocalized with p-SMAD2/3 (red) (Figure 3C). These findings prompted us to assume that SRPX2 may be induced by TGF-β/SMADs signaling. To address this hypothesis, HPFs were treated with SB-431542 (a selective inhibitor of TGFβR1) and SIS3-HCl (a specific inhibitor of p-SMAD3), followed by TGF-β1 induction, respectively. Of important noted, the inhibition of TGFβR1 by SB-431542 (Figure 3D-E) and p-SMAD3 by SIS3-HCl (Figure 3F-G) could both significantly abolish the upregulation of SRPX2 induced by TGF-β1, accompanied by attenuated FMT related proteins, indicating that TGFβR1 and SMAD3 were indispensable factors for the induction of SRPX2 during FMT. Altogether, these results corroborated that the overexpression of SRPX2 during FMT was induced by TGF-β1 in a TGFβRI/SMAD3-dependent manner.

### SRPX2 enhanced the phosphorylation of SMAD2/3 through the SRPX2/AP1/SMAD7 axis in the process of TGF-β1-induced FMT

**Figure 4:**
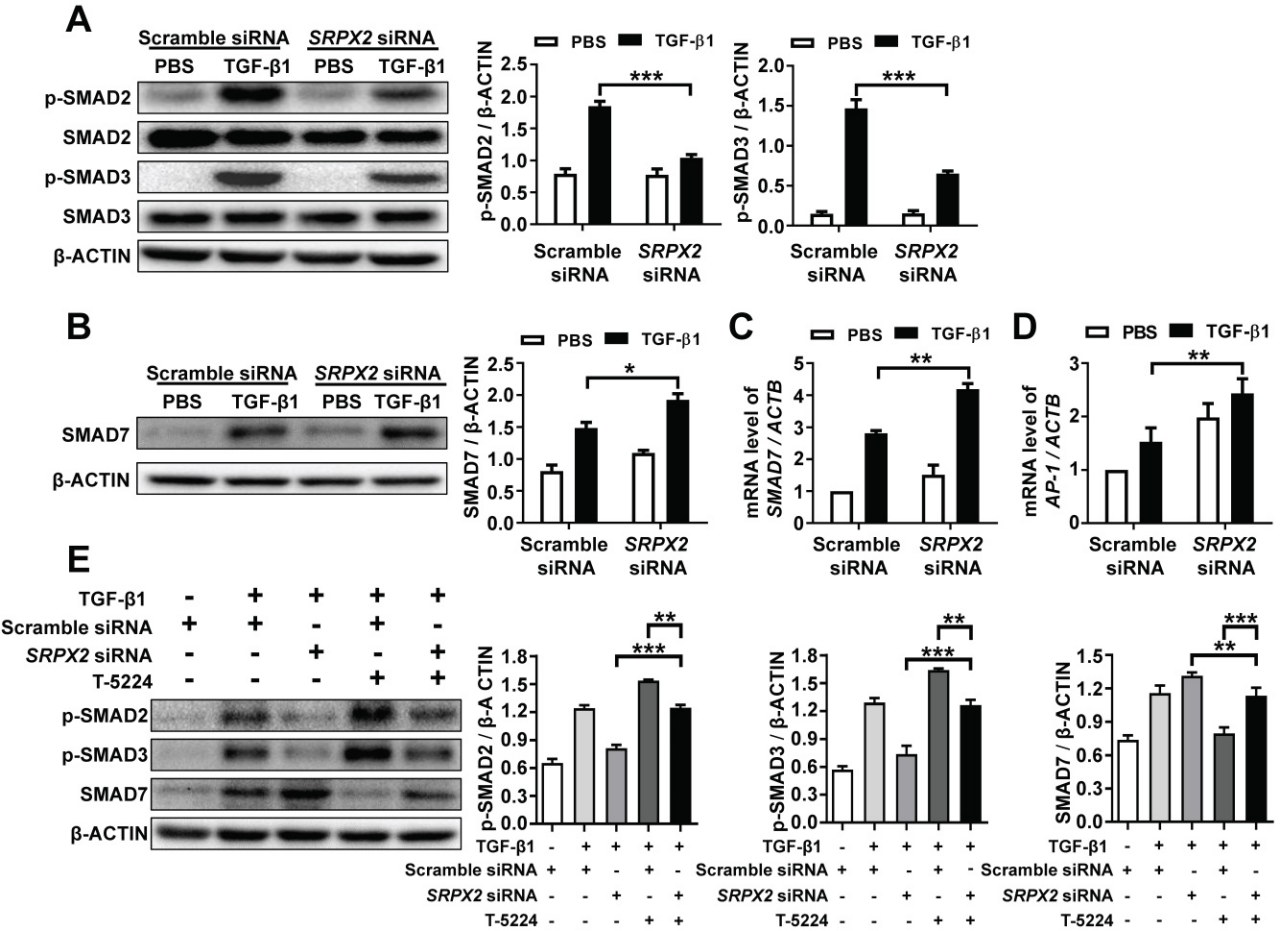
** SRPX2 regulated TGF-β/SMADs signaling pathways by AP1 and SMAD7. A:** Results for Western blot analysis of p-SMAD2, SMAD2, p-SMAD3 and SMAD3 expression in HPFs following TGF-β1 stimulation. **B-C**: Western blot** (B)** and RT-PCR** (C)** analysis of SMAD7 expression in HPFs following TGF-β1 induction. **D**: Expression of AP1 in HPFs after TGF-β1 stimulation. **E**: Western blot results for analysis of the levels of P-SMAD2, P-SMAD3 and SMAD7 in HPFs pre-treated with T-5224 (an inhibitor for AP-1) treatment following TGF-β1 induction. The data are represented as the mean ± SEM of three independent experiments. *, p < 0.05; **, p < 0.01; ***, p < 0.001.

It has been demonstrated that SRPX2 was up-regulated by the stimulation of TGF-β1, which was critical for FMT. However, it is still masked how SRPX2 affects FMT process. To address the process in detail, firstly, the impact of SRPX2 on the phosphorylation of SMAD2 and SMAD3 was detected in TGF-β1-treated fibroblasts. Impressively, the suppression of SRPX2 in HPFs dramatically blocked the phosphorylation of SMAD2 and SMAD3 induced by TGF-β1 (Figure 4A). Given that SMAD7 has been identified as an intracellular antagonist for SMAD2/3 phosphorylation 26, we further explored whether the blockade of SMAD2/3 phosphorylation in *SRPX2*-siRNA-treated HPFs was mediated by SMAD7. Indeed, both Western blot and RT-PCR results exhibited an overexpression of SMAD7 when SRPX2 was blunt in HPFs (Figure 4B and C), suggesting SRPX2 was in the upstream of SMAD7. Moreover, it has been reported that AP-1, a transcription factor for SMAD7, may be a target for the expression of SRPX2 27, 28, prompting us to assume a SRPX2/AP1/SMAD7 axis exists within the TGF-β1-induced FMT process. To prove the assumption, the expression of AP-1 in HPFs was detected. Remarkably, the silence of SRPX2 enhanced the expression of AP-1 in TGF-β1 treated HPFs (Figure 4D). Next, a rescue experiment was conducted to reinforce the conception. With the treatment of T-5224, a selective inhibitor for AP-1, the up-regulation of SMAD7 by *SRPX2* siRNA was abrogated, together with the increased phosphorylation of SMAD2/3 (Figure 4E), indicating that SMAD2/3 phosphorylation was regulated by SRPX2/AP1/SMAD7 axis. Together, all above data suggested that SPRX2 could regulate SMAD2/3 phosphorylation in the TGF-β1-induced FMT process through the SRPX2/AP1/SMAD7 axis, which formed a positive feedback loop within the TGF-β signaling pathway.

### *Srpx2* siRNA loaded liposomes were prepared for the treatment of pulmonary fibrosis

**Figure 5:**
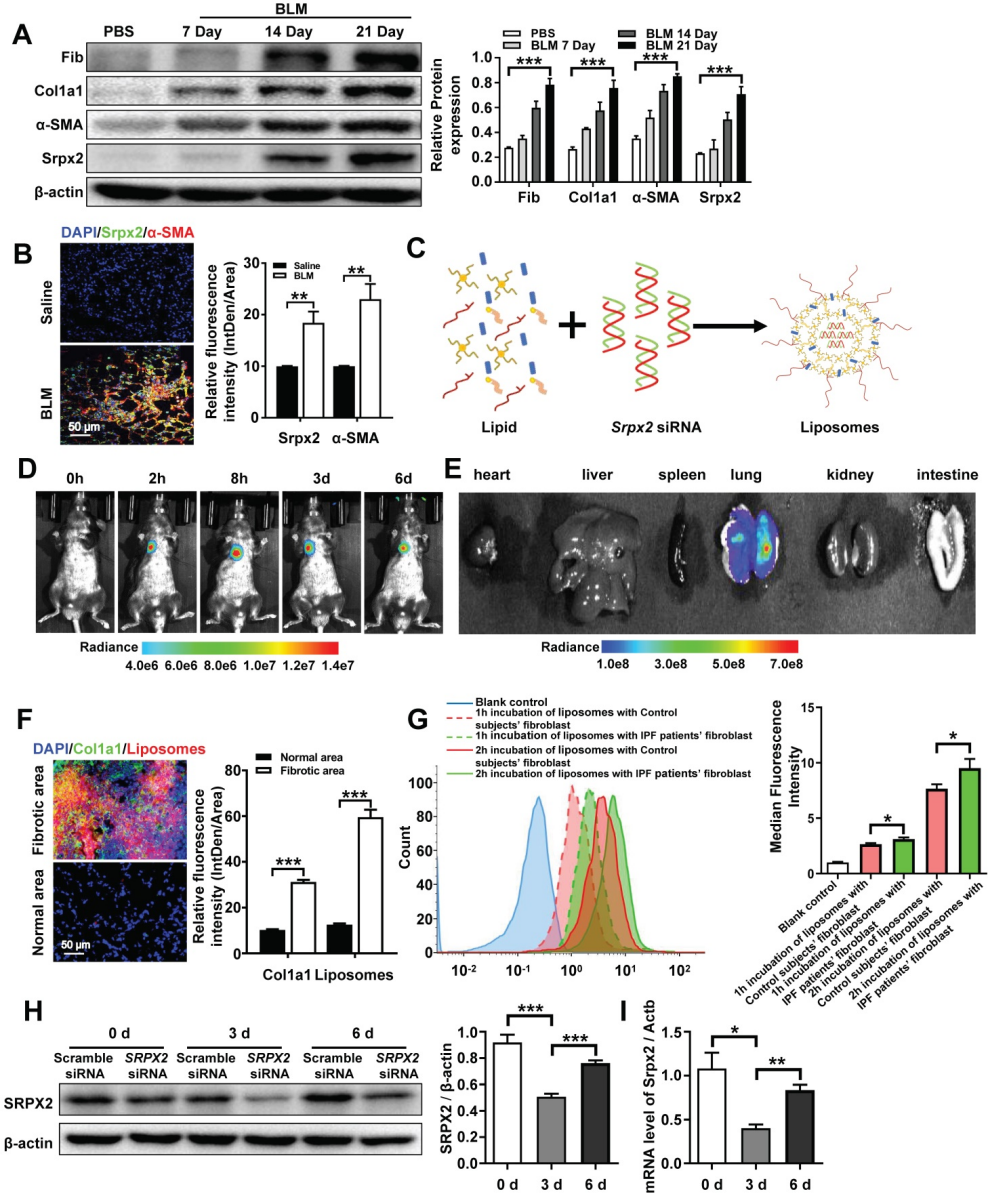
** Biodistribution of the liposomes after intratracheal injection. A**: Western blot analysis of Srpx2, fibronectin, Col1a1 and α-SMA expression in the lungs of mice following BLM induction. Three mice were included in each study group. **B**: Results for co-immunostaining of Srpx2 and α-SMA in BLM-induced lung sections. **C**: Schematic diagram showing the preparation of *Srpx2* siRNA-loaded liposomes. **D:** Representative IVIS images of a mouse at different time points after the administration of DiR-labeled liposomes.** E:** *Ex vivo* fluorescence images of major organs from mice. **F**: Immunofluorescence image showing the biodistribution of DiI-labeled liposomes (red) and Col1a1 (green) in the lungs of BLM-induced mice. The nuclei were stained blue by DAPI, and the images were taken at ×400 magnification. **G**: phagocytosis of *SRPX2* siRNA-loaded liposomes in fibroblasts from IPF patients and control subjects. **H-I**: Western blot** (H)** and RT-PCR** (I)** analysis of the levels of temporal changes in Srpx2 expression in the lungs of transfected mice after BLM induction. Three mice were included in each study group. The data are represented as the mean ± SEM. *, p < 0.05; **, p < 0.01; ***, p < 0.001.

Based on the mechanistic studies, we attempted to move the concept to therapeutic application. Similar with the data obtained from IPF patients (Figure 1D), the overexpression of Srpx2 was confirmed in BLM-induced pulmonary fibrosis mouse model in a time-depend manner, along with the high expressions of fibrotic markers (Figure 5A). Specifically, Srpx2 mainly concentrated on fibrotic foci (Figure 5B). Then, cationic liposome was prepared for the delivery of *Srpx2* siRNA to pulmonary fibrosis mice (Figure 5C). As characterized, the siRNA loaded liposomes possess a uniform size distribution around 102 nm (PDI = 0.07) and its zeta-potential approached to electric neutrality (2.8 mv) due to the electronegative nature of siRNA (Supplementary Figure 3A). Additionally, the liposomes could efficiently encapsulate siRNA *via* electrostatic interactions with a high entrapment efficiency of 95% (Supplementary Figure 3A). The transmission electron microscopy (TEM) images indicated a uniform sphere morphology of the liposomes (Supplementary Figure 3B). Moreover, the liposomes could maintain its stability for at least 24 hours (Supplementary Figure 3C and D). Additionally, the favorable biocompatibility of the siRNA-loaded liposomes was confirmed by CCK8 assay (Supplementary Figure 4A). To figure out the biodistribution of the liposomes in the mice, the DiR-loaded liposomes were prepared and administrated to mice by intratracheally. Then, the mice were surveyed and recorded by IVIS at different time points (0 h, 2 h, 8 h, 3 d, 6 d). The fluorescence signal was predominantly accumulated in the lung and gradually weakened over time (Figure 5D). Indeed, the *ex vivo* images (Figure 5E) indicated that intratracheal injection of liposomes concentrated in the lung rather than in other organs. To address the biodistribution of the liposomes in lung from pulmonary fibrosis mice, DiI-labeled liposomes were used. Notedly, majority of the liposomes (red) located in the fibrotic zone marked by enriched collagen I (green), indicating an abundant uptake of the liposomes by fibroblasts (Figure 5F). In line with above results, the fibroblasts originated from IPF patients also exhibited an enhanced uptake of liposomes than that from control subjects, which might be attributed to the strengthened phagocytosis capability of myofibroblasts (Figure 5G). Finally, the *in vivo* inhibition efficiency for Srpx2 was assessed after the administration of the liposomes. The lowest Srpx2 expression was observed on day 3 after the intratracheal administration of the siRNA-loaded liposomes and, excitingly, the liposomes-based therapeutic repressed Srpx2 expression for at least 6 days (Figure 5 H and I). It is noted that some nanoparticles can lead to acute toxic effects, and even, causing a systemic inflammatory response 29. To address this concern, we performed *in vivo* study to assess the safety of liposomes in mice. Of note, intratracheal administration of liposomes neither lead to the inflammation of lung and epithelial apoptosis nor toxicity to heart, liver, spleen, kidney, and intestinal tract (Supplementary Figure 4B-D), indicating that intratracheal injection of liposomes were well tolerated and safe in mice.

Taken together, the above results verify that the liposomes selectively accumulated in the pulmonary fibrotic lesion and efficiently suppressed the expression of Srpx2 in lung with a good security, which could be a promising therapeutic for pulmonary fibrosis.

### Intratracheal administration of *Srpx2* siRNA loaded liposomes exhibited excellent antifibrosis effects on BLM-induced pulmonary fibrosis

**Figure 6:**
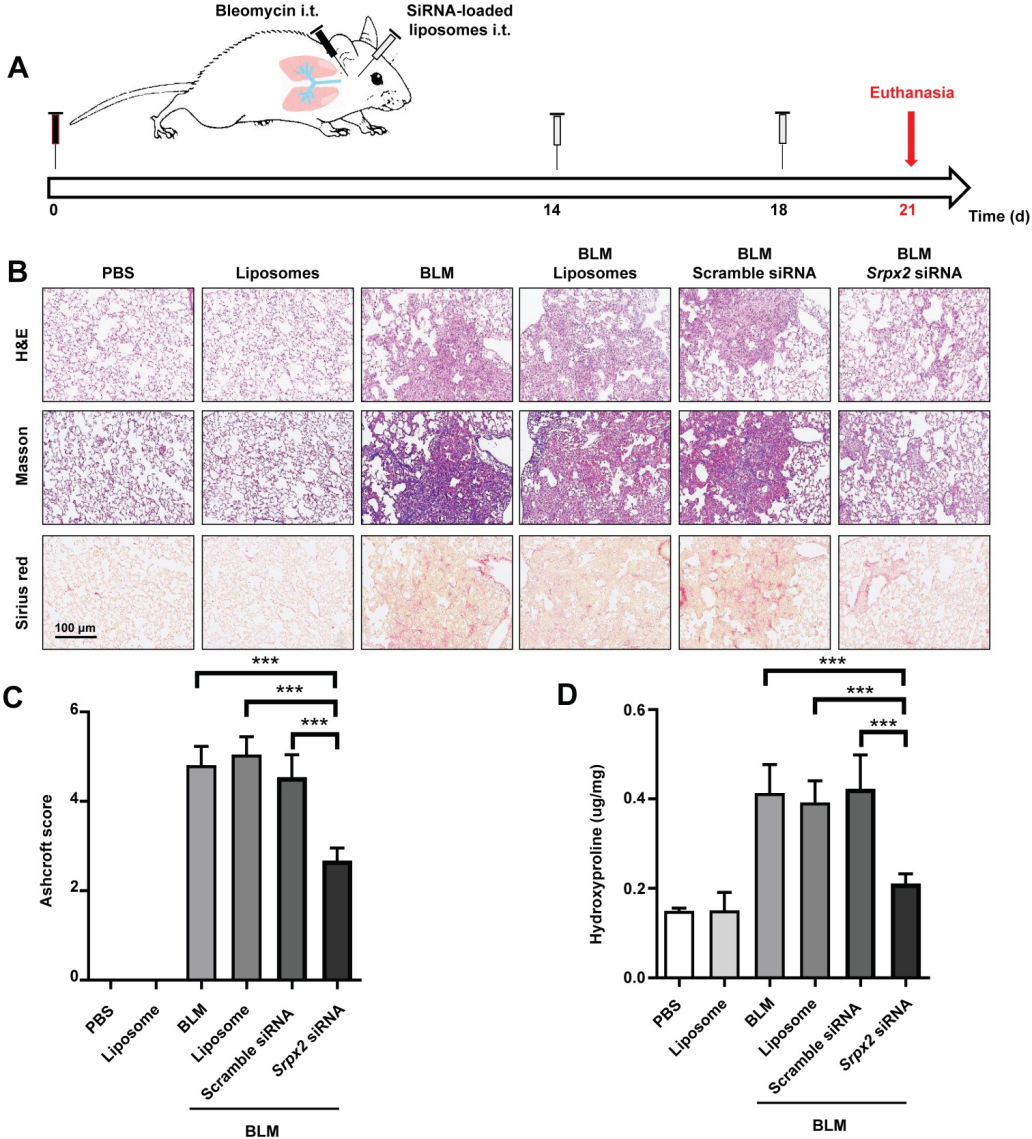
***Srpx2* siRNA-loaded liposomes protected mice from BLM-induced lung injury and fibrosis. A**: Schematic for experimental design and time course of BLM-treated WT mice administered with either Scrambled or *Srpx2* siRNA-loaded liposomes. **B**: Histological analysis of the severity of lung fibrosis in mice after BLM induction with Scrambled or *Srpx2* siRNA-loaded liposomes. Left panel: representative images for H&E (top), Masson staining (middle) and Sirius red (bottom). Images were captured at ×200 magnification. **C**: A bar graph showed the quantitative mean score of the severity of fibrosis. **D**: Quantification of hydroxyproline in BLM-induced mice. Six mice were included in each study group. The data are represented as the mean ± SEM. ***, p < 0.001.

The therapeutic effects of *Srpx2* siRNA loaded liposomes were assessed in mice following BLM induction for 21 days. C57BL/6 mice were intratracheally administered liposomes loaded with scrambled or *Srpx2* siRNA (dosage of siRNA, 1 mg/kg) on day 14 and day 18 (Figure 6A). Significantly attenuated lung injury and pulmonary fibrosis were observed in the *Srpx2* siRNA-loaded liposomes group, as evidenced by the H&E, Masson's trichrome and Sirius red staining (Figure 6B). In particular, Ashcroft scores, assessing the severity of pulmonary fibrosis, were substantially decreased in *Srpx2* siRNA-loaded liposomes group (Figure 6C). Consistently, the hydroxyproline content in the lung homogenates, a marker correlated with fibrosis severity, was much lower in* Srpx2* siRNA-loaded liposomes treated mice than that in scrambled siRNA group (Figure 6D).

**Figure 7:**
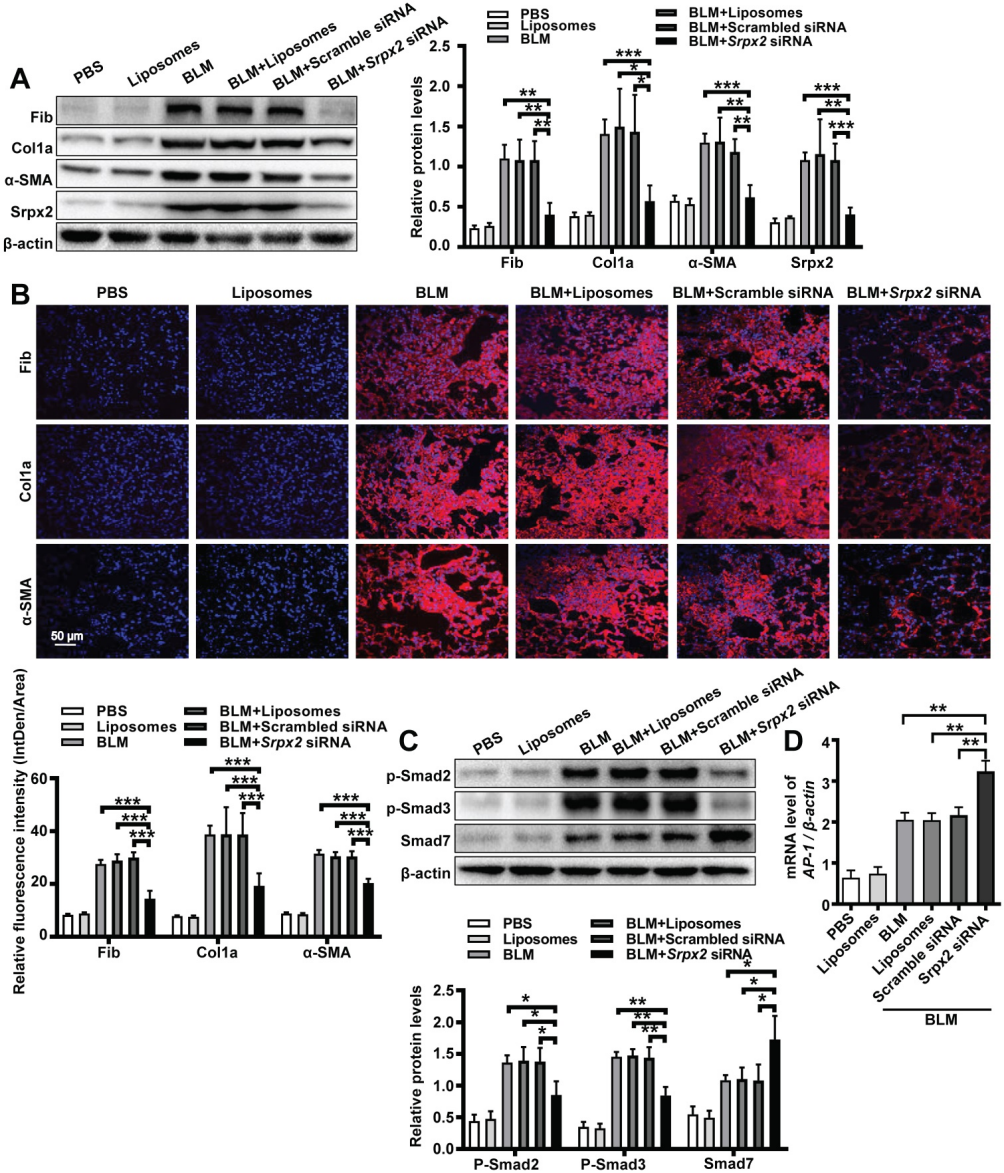
** Srpx2 promoted FMT in BLM-induced pulmonary fibrosis. A**: Western blot analysis of Fibronectin, Col1a1, α-SMA and Srpx2 expression in mice after BLM induction with Scrambled or *Srpx2* siRNA-loaded liposomes. **B**: Representative images of immunostaining of Fibronectin, Col1a1 and α-SMA in the mice lung sections. The nuclei were stained blue by DAPI, and the images were taken under original magnification ×400. **C**: Western blot analysis of p-Smad2, p-Smad3 and Smad7 expression in mice after BLM induction. **D**: RT-PCR analysis of *AP-1* expression in mice in each group. Six mice were included in each study group. The data are represented as the mean ± SEM. *, p < 0.05; **, p < 0.01; ***, p < 0.001.

To further evaluate the therapeutic effect of the liposomes, we next conducted Western blot, RT-PCR and immunostaining to examine the fibrotic markers in the lung. Consistent to above results, mice administered with *Srpx2* siRNA-loaded liposomes displayed attenuated expressions of fibronectin, collagen I and α-SMA (Figure 7A and B, Supplementary Figure 5). In line with the *in vitro* studies (Figure 4), the *in vivo* studies also indicated that suppression of *Srpx2* in lung could effectively repress the phosphorylation of Smad2/3, coupled with a dramatical overexpression of Smad7 and AP-1 (Figure 7C and D). Together, our data support the notion that intratracheal injection of *Srpx2* siRNA-loaded liposomes could be a potent therapeutic approach against pulmonary fibrosis *via* blocking attenuated FMT.

## Discussion

**Figure 8:**
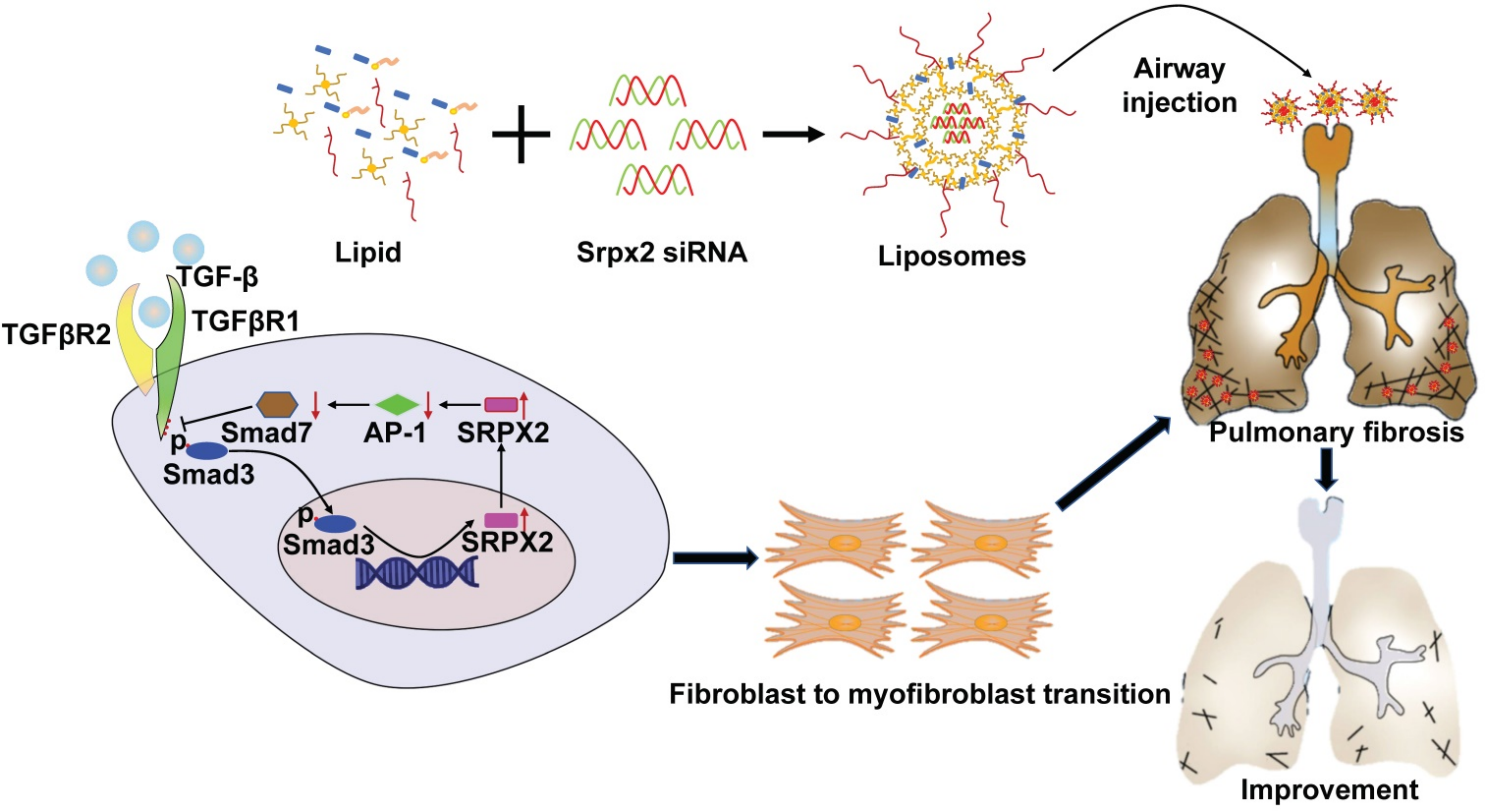
** A diagram for mechanisms underlying SRPX2 regulation of pulmonary fibrosis.** Specifically, TGF-β enhanced SRPX2 expression in a TGFβR1 and SMAD3-dependent manner. Subsequently, SRPX2 inhibited the expression of AP-1, by which it blunt SMAD7 expression and accelerate the phosphorylation of SMAD2/3. The activated TGF-β/SMADs signaling would promote the FMT and pulmonary fibrosis.

IPF is a chronic and progressive interstitial lung disease of unknown origin with an average life expectancy of 2-3 years after diagnosis 30. Until now, no treatment except lung transplantation is able to halt or reverse the process of IPF 31. Although two drugs, pirfenidone and nintedanib, could relieve the disease progression, neither drug improves or even maintains lung function 32. Besides, both of them exhibit severe side effects. Therefore, developing a novel effective treatment for IPF is urgently needed. Herein, we demonstrated that SRPX2 was overexpressed in lungs originated from IPF patients and pulmonary fibrosis mice. Specifically, SRPX2 was over-expressed in fibroblasts. Mechanistic studies revealed a TGFβR1/SMAD3/SRPX2/AP-1/SMAD7 positive feedback regulatory loop. In detail, TGF-β enhanced SRPX2 expression in a TGFβR1 and SMAD3-dependent manner. And then, SRPX2 selectively inhibited the expression of AP-1, which could subsequently blunt SMAD7 expression. low level of SMAD7 in turn accelerated the phosphorylation of SMAD2/3 to promote the proliferation, migration and transition of fibroblasts, thereby exacerbating pulmonary fibrosis. In line with this observation, intratracheal injection of liposomes carrying *Srpx2* siRNA reversed BLM-induced lung injury and fibrosis (Figure 8). Collectively, these results not only raise novel insights into the understanding of the role of SRPX2 underlying IPF, but also provide a potent therapeutic approach to improve IPF.

Previous studies have demonstrated that SRPX2 could efficaciously regulate proliferation, migration, differentiation and apoptosis of tumor cells *via* different signaling pathways 11, 12, 28, 33. Surprisingly, it was noted that the differential expression of Srpx2 family genes, including Srpx2, during the process of FMT were outstanding in the data of deep RNA-Seq. To confirm this data, the expression of SRPX2 was also detected in fibroblasts originated from IPF patients' and control subjects' lung. Indeed, an overexpression of SRPX2 was observed in IPF patients derived fibroblasts, along with up-regulated fibrotic markers. Consistently, similar data were illustrated in the lungs of IPF patients and pulmonary fibrosis mice. These observations prompted us to explore the effects of SRPX2 in fibroblasts.

Myofibroblasts are critical in the process of pulmonary fibrosis by their secretion of ECM proteins, leading to tissue stiffness and respiratory failure 3, 34. Considering the pivotal role of myofibroblast in pulmonary fibrosis, we firstly examined the impact of SRPX2 on FMT. Actually, knocking-down SRPX2 robustly abrogated the process of FMT, as evidenced by attenuated myofibroblasts related proteins. Of most concern, the repression of SRPX2 reversed IPF patient's lung fibroblasts undergoing FMT in a dose-depend manner. In addition, we demonstrated that silencing SRPX2 could also attenuate the proliferation and migration of fibroblasts, which are believed to be involved in the pathogenesis of pulmonary fibrosis. To our best knowledge, this is the first report of SRPX2 contribution in controlling fibroblast behaviors.

Another critical issue is the underlying mechanisms by which SRPX2 regulated the transition, proliferation and migration of fibroblasts. It is compelling noted that TGF-β1 is one of the most potent and well-studied inducers for FMT 35. Within the activation of TGF-β signaling pathway, TGF-β1 dimer firstly binds to TGFβR2, outside the cell membrane of the fibroblasts. Next, TGFβR1 is recruited into the complex and phosphorylated by TGFβR2, and then the intracellular signaling pathway is transmitted through canonical pathway (Smad proteins) or non-canonical pathways (MAPKs and Rho family members) 9, 10. Indeed, TGF-β1 induced the expression of SRPX2 in a dose and time-depend manner. Of important note, the overexpressed SRPX2 in fibrotic condition was dependent on the canonical pathway. Particularly, TGFβR1 and SMAD3 were identified as the essential mediators. Interestingly, we fund that the reduction of SRPX2 could significantly suppress the phosphorylation of SMAD2 and SMAD3 induced by TGF-β1.

To address the notion that SRPX2 regulated the TGF-β/SMADs signaling pathway, we next examined the expression of SMAD7, which interferes with the phosphorylation of SMAD2 and SMAD3 *via* binding with activated TGFβR1 26. As expected, knocking-down SRPX2 by siRNA robustly enhanced the expression of SMAD7. It was noted that downregulation of SRPX2 accelerated YAP phosphorylation, resulting in a reduced nuclear translocation of YAP 28. Meanwhile, the nuclear-translocated YAP participated in the transcription of AP-1, substantially reduced SMAD7 promoter reporter activity 27. In line with these results, we demonstrated that SRPX2 repressed the expression of SMAD7 *via* AP-1 depend manner, as evidenced by inhibiting AP-1 reversed the overexpression of SMAD7 induced by SRPX2 siRNA and activated TGF-β/SMADs signaling pathway. Additionally, similar data were also obtained in the following *in vivo* studies. Collectively, SRPX2 was upregulated by TGF-β1 in a TGFβR1/SMAD3-dependent manner. After that, the elevated SRPX2 would inhibit the expression of AP1, and subsequently down-regulated the expression of SMAD7, leading to an enhanced TGF-β/SMADs signaling pathway. This positive feedback loop centered on SRPX2 would further promote fibroblasts differentiating into myofibroblasts, exacerbating pulmonary fibrosis.

Although numerous clinical trials have been carried out to characterize viable drugs for IPF, unfortunately, no effective therapeutic approach was currently available to cure or even halt the progression of IPF 31. Previous studies 30, 36, including our 15, have illustrated that liposomes loading with siRNA are established as drug carriers for airway injection owing to their safety and effectiveness to provide controlled drug release in the lung. Inspiringly, patisiran, the first siRNA drug based on liposomes, was approved by the FDA in 2018, moving the concept to clinical settings 37. The most exciting discovery in this report is that liposomes carrying the *Srpx2* siRNA could be efficiently phagocytized by the fibroblasts in fibrotic lesions of the lung following intratracheal injection and suppress the expression of Srpx2 for at least 6 days. More importantly, *Srpx2* siRNA-loaded liposomes was intratracheally injected into mice during the “fibrotic” phase of the model, which is more applicable and reflective to the clinical management of IPF patients. Since our previous work 15 demonstrated that liposomes maybe uptake by macrophages and regulate macrophages polarization, we also assessed the impact of *Srpx2* siRNA-loaded liposomes on macrophages. No obvious difference was detected on the polarization of alternative activated macrophages, which were one of major sources of TGF-β 1 (Supplementary Figure 6A). Additionally, the expression of Srpx2 also showed no difference following IL-4 stimulated macrophages (Supplementary Figure 6B), indicating that the protection of *Srpx2* siRNA-loaded liposomes on pulmonary fibrosis is duo to suppression of fibroblasts behavior, not macrophages activation. Overall, intratracheal injection of *Srpx2* siRNA loaded liposomes could block TGF-β signaling pathway and subsequently decelerate the process of FMT, contributing to the protection of mice from BLM-induced lung injury and fibrosis, which provide an available, safe and effective therapeutic approach for IPF.

In conclusion, we firstly demonstrated that SRPX2 was overexpressed in the fibroblasts originated from IFP patients and pulmonary fibrosis mice. Mechanistically, SRPX2 was upregulated by TGF-β1 canonical signaling pathway during the process of FMT. In addition, the elevated SRPX2 would inhibit the expression of AP1, and then attenuated SMAD7 expression, forming a positive feedback loop to enhance TGF-β/SMADs signaling, which could finally promote FMT and exacerbate pulmonary fibrosis. Inspiringly, administration of liposomes carrying *Srpx2* siRNA significantly reduced the expression of Srpx2 and then suppressed FMT, contributing to the improvement of the pulmonary fibrosis induced by BLM. Briefly, our data supported that intratracheal administration of *Srpx2* siRNA loaded liposomes could be a promising therapeutic approach against pulmonary fibrosis in clinical settings.

## Supplementary Material

Supplementary figures.

## Figures and Tables

**Table 1:** Characteristics of subjects from which lung samples were taken

	Lung samples	
	IPF (n = 6)	Control (n = 6)
Age (years)	63.50 ± 11.79	58.67 ± 5.64
BMI	23.83 ± 3.80	23.05 ± 1.14
**Sex**		
Female	3 (50.00%)	3 (50.00%)
Male	3 (50.00%)	3 (50.00%)
**FVC**		
Percent Predicted	76.09 ± 15.24	NA
DLCO	48.05 ± 6.30	NA

BMI: body mass index; FVC: forced vital capacity; DLCO: diffusion capacity for carbon monoxide.

## References

[1] Rao LZ, Wang Y, Zhang L, Wu G, Zhang L, Wang FX (2020). IL-24 deficiency protects mice against bleomycin-induced pulmonary fibrosis by repressing IL-4-induced M2 program in macrophages. Cell death and differentiation.

[2] Raghu G, Behr J, Brown KK, Egan JJ, Kawut SM, Flaherty KR (2013). Treatment of idiopathic pulmonary fibrosis with ambrisentan: a parallel, randomized trial. Annals of internal medicine.

[3] Hettiarachchi SU, Li YH, Roy J, Zhang F, Puchulu-Campanella E, Lindeman SD (2020). Targeted inhibition of PI3 kinase/mTOR specifically in fibrotic lung fibroblasts suppresses pulmonary fibrosis in experimental models. Sci Transl Med.

[4] Liu P, Miao K, Zhang L, Mou Y, Xu Y, Xiong W (2020). Curdione ameliorates bleomycin-induced pulmonary fibrosis by repressing TGF-beta-induced fibroblast to myofibroblast differentiation. Respir Res.

[5] Wang Y, Zhang L, Wu GR, Zhou Q, Yue H, Rao LZ (2021). MBD2 serves as a viable target against pulmonary fibrosis by inhibiting macrophage M2 program. Science advances.

[6] Wolters PJ, Collard HR, Jones KD (2014). Pathogenesis of idiopathic pulmonary fibrosis. Annu Rev Pathol.

[7] Tao L, Bei Y, Chen P, Lei Z, Fu S, Zhang H (2016). Crucial Role of miR-433 in Regulating Cardiac Fibrosis. Theranostics.

[8] Hata A, Chen YG (2016). TGF-beta Signaling from Receptors to Smads. Cold Spring Harbor perspectives in biology.

[9] Lee TH, Yeh CF, Lee YT, Shih YC, Chen YT, Hung CT (2020). Fibroblast-enriched endoplasmic reticulum protein TXNDC5 promotes pulmonary fibrosis by augmenting TGFbeta signaling through TGFBR1 stabilization. Nat Commun.

[10] Derynck R, Zhang YE (2003). Smad-dependent and Smad-independent pathways in TGF-beta family signalling. Nature.

[11] Gao Z, Wu J, Wu X, Zheng J, Ou Y (2020). SRPX2 boosts pancreatic cancer chemoresistance by activating PI3K/AKT axis. Open medicine.

[12] Li X, Liu J, Sun H, Zou Y, Chen J, Chen Y (2020). SRPX2 promotes cell proliferation and invasion via activating FAK/SRC/ERK pathway in non-small cell lung cancer. Acta biochimica Polonica.

[13] Dong Y, Siegwart DJ, Anderson DG (2019). Strategies, design, and chemistry in siRNA delivery systems. Advanced drug delivery reviews.

[14] Zheng M, Tao W, Zou Y, Farokhzad OC, Shi B (2018). Nanotechnology-Based Strategies for siRNA Brain Delivery for Disease Therapy. Trends in biotechnology.

[15] Pan T, Zhou Q, Miao K, Zhang L, Wu G, Yu J (2021). Suppressing Sart1 to modulate macrophage polarization by siRNA-loaded liposomes: a promising therapeutic strategy for pulmonary fibrosis. Theranostics.

[16] Love KT, Mahon KP, Levins CG, Whitehead KA, Querbes W, Dorkin JR (2010). Lipid-like materials for low-dose, *in vivo* gene silencing. Proc Natl Acad Sci U S A.

[17] Raghu G, Collard HR, Egan JJ, Martinez FJ, Behr J, Brown KK (2011). An official ATS/ERS/JRS/ALAT statement: idiopathic pulmonary fibrosis: evidence-based guidelines for diagnosis and management. American journal of respiratory and critical care medicine.

[18] Hu Y, Yu J, Wang Q, Zhang L, Chen X, Cao Y (2020). Tartrate-Resistant Acid Phosphatase 5/ACP5 Interacts with p53 to Control the Expression of SMAD3 in Lung Adenocarcinoma. Molecular therapy oncolytics.

[19] Miao K, Zhang L, Pan T, Wang Y (2020). Update on the role of endoplasmic reticulum stress in asthma. American journal of translational research.

[20] Yao Y, Wang Y, Zhang Z, He L, Zhu J, Zhang M (2016). Chop Deficiency Protects Mice Against Bleomycin-induced Pulmonary Fibrosis by Attenuating M2 Macrophage Production. Mol Ther.

[21] Miao K, Pan T, Mou Y, Zhang L, Xiong W, Xu Y (2020). Scutellarein inhibits BLM-mediated pulmonary fibrosis by affecting fibroblast differentiation, proliferation, and apoptosis. Ther Adv Chronic Dis.

[22] Wang Y, Zhu J, Zhang L, Zhang Z, He L, Mou Y (2017). Role of C/EBP homologous protein and endoplasmic reticulum stress in asthma exacerbation by regulating the IL-4/signal transducer and activator of transcription 6/transcription factor EC/IL-4 receptor alpha positive feedback loop in M2 macrophages. The Journal of allergy and clinical immunology.

[23] Pan T, Zhang L, Miao K, Wang Y (2020). A crucial role of endoplasmic reticulum stress in cellular responses during pulmonary arterial hypertension. American journal of translational research.

[24] Zhang H, Zhou P, Wei Y, Yue H, Wang Y, Hu M (2020). Histopathologic Changes and SARS-CoV-2 Immunostaining in the Lung of a Patient With COVID-19. Annals of internal medicine.

[25] Xie N, Tan Z, Banerjee S, Cui H, Ge J, Liu RM (2015). Glycolytic Reprogramming in Myofibroblast Differentiation and Lung Fibrosis. American journal of respiratory and critical care medicine.

[26] Nakao A, Fujii M, Matsumura R, Kumano K, Saito Y, Miyazono K (1999). Transient gene transfer and expression of Smad7 prevents bleomycin-induced lung fibrosis in mice. The Journal of clinical investigation.

[27] Qin Z, Xia W, Fisher GJ, Voorhees JJ, Quan T (2018). YAP/TAZ regulates TGF-beta/Smad3 signaling by induction of Smad7 via AP-1 in human skin dermal fibroblasts. Cell Commun Signal.

[28] Wu Z, Wang C, Chen Y, Sun Z, Yan W (2020). SRPX2 Promotes Cell Proliferation and Invasion in Osteosarcoma Through Regulating Hippo Signaling Pathway. Onco Targets Ther.

[29] Park EJ, Kim SN, Yoon C, Cho JW, Lee GH, Kim DW (2021). Repeated intratracheal instillation of zinc oxide nanoparticles induced pulmonary damage and a systemic inflammatory response in cynomolgus monkeys. Nanotoxicology.

[30] Hou J, Ji Q, Ji J, Ju S, Xu C, Yong X (2021). Co-delivery of siPTPN13 and siNOX4 via (myo)fibroblast-targeting polymeric micelles for idiopathic pulmonary fibrosis therapy. Theranostics.

[31] George PM, Patterson CM, Reed AK, Thillai M (2019). Lung transplantation for idiopathic pulmonary fibrosis. The Lancet Respiratory medicine.

[32] Wells AU, Flaherty KR, Brown KK, Inoue Y, Devaraj A, Richeldi L (2020). Nintedanib in patients with progressive fibrosing interstitial lung diseases-subgroup analyses by interstitial lung disease diagnosis in the INBUILD trial: a randomised, double-blind, placebo-controlled, parallel-group trial. The Lancet Respiratory medicine.

[33] Salmi M, Bruneau N, Cillario J, Lozovaya N, Massacrier A, Buhler E (2013). Tubacin prevents neuronal migration defects and epileptic activity caused by rat Srpx2 silencing in utero. Brain.

[34] Hinz B, Lagares D (2020). Evasion of apoptosis by myofibroblasts: a hallmark of fibrotic diseases. Nature reviews Rheumatology.

[35] Tomcik M, Palumbo-Zerr K, Zerr P, Sumova B, Avouac J, Dees C (2016). Tribbles homologue 3 stimulates canonical TGF-beta signalling to regulate fibroblast activation and tissue fibrosis. Annals of the rheumatic diseases.

[36] da Silva AL, de Oliveira GP, Kim N, Cruz FF, Kitoko JZ, Blanco NG (2020). Nanoparticle-based thymulin gene therapy therapeutically reverses key pathology of experimental allergic asthma. Science advances.

[37] Heras-Palou C (2019). Patisiran's path to approval as an RNA therapy. Nature.

