# Local administration of liposomal-based Srpx2 gene therapy reverses pulmonary fibrosis by blockading fibroblast-to-myofibroblast transition: Erratum

**DOI:** 10.7150/thno.74512

**Published:** 2022-07-08

**Authors:** Qi Wang, Juan Liu, Yinan Hu, Ting Pan, Yongjian Xu, Jun Yu, Weining Xiong, Qing Zhou, Yi Wang

**Affiliations:** 1 Department of Respiratory and Critical Care Medicine, Key Laboratory of Pulmonary Diseases of National Health Commission, Key Site of National Clinical Research Center for Respiratory Disease, Wuhan Clinical Medical Research Center for Chronic Airway Diseases, Tongji Hospital, Tongji Medical College, Huazhong University of Sciences and Technology, 1095 Jiefang Ave, Wuhan 430030, China.; 2 Department of Pulmonary and Critical Care Medicine, Center of Respiratory Medicine, China-Japan Friendship Hospital, 100029, Beijing, China.; 3 Department of Thoracic Surgery, Tongji Hospital, Tongji Medical College, Huazhong University of Sciences and Technology, 1095 Jiefang Ave, Wuhan 430030, China.; 4 Department of Respiratory and Critical Care Medicine, Shanghai Key Laboratory of Tissue Engineering, Shanghai Ninth People's Hospital, Shanghai Jiaotong University School of Medicine, 639 Zhizaoju Lu, Shanghai, 200011, China.; 5 The Center for Biomedical Research, Tongji Hospital, Tongji Medical College, Huazhong University of Sciences and Technology, 1095 Jiefang Ave, Wuhan 430030, China.

In the original publication, error was found in Fig 7B. During the assembling of figure, in Fig 7B, we mistakenly put the same image of Col1a1 immunostaining within the Liposomes group into the PBS group. The correct figure is shown below. The authors confirm that these corrections do not change the result interpretation or conclusions of the article. The authors are deeply sorry and sincerely apologize for any inconvenience or misunderstanding that may have caused.

## Figures and Tables

**Fig 7:**
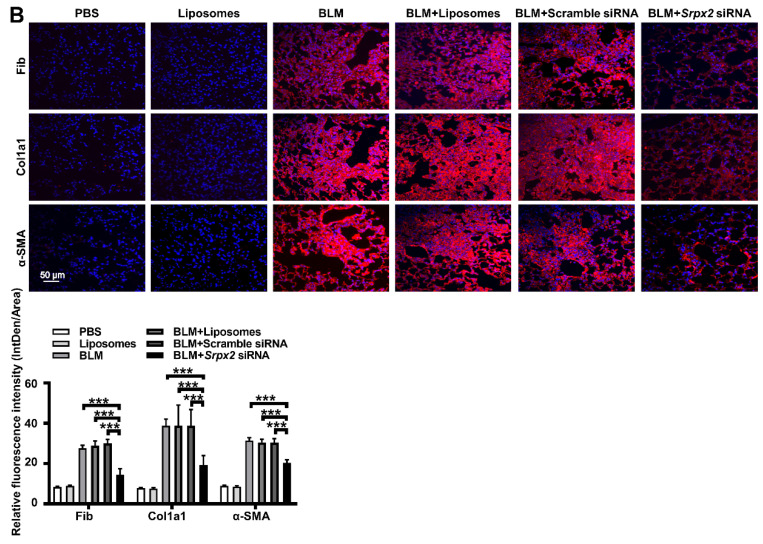
** B:** Representative images of immunostaining of Fibronectin, Col1a1 and α-SMA in the mice lung sections. The nuclei were stained blue by DAPI, and the images were taken under original magnification ×400.

