# Supplementary material for: Local administration of liposomal-based Srpx2 gene therapy reverses pulmonary fibrosis by blockading fibroblast-to-myofibroblast transition
Source: Theranostics. 2021 May 13;11(14):7110–25. doi: 10.7150/thno.61085 (PMC8171094; doi:10.7150/thno.61085)
Supplement: Supplementary file 1 — Supplementary figures. [file thnov11p7110s1.pdf]

Supplemental Figure 1. Representative images of immunostaining of FIBRONECTIN, COL1A1 and α-SMA in HPFs. The nuclei were stained blue by DAPI, and the images were taken under original magnification ×400. The data are represented as the mean ± SEM of three independent experiments. *, $p < 0.05$; **, $p < 0.01$; ***, $p < 0.001$.

Supplemental Figure 2. A: Representative results for Transwell assay in *SRPX2* siRNA or Scrambled siRNA treated HPFs. **B**: Representative results for EdU staining in *SRPX2* siRNA or Scrambled siRNA treated HPFs. The data are represented as the mean ± SEM of three independent experiments. *, $p < 0.05$; ***, $p < 0.001$.

Supplemental Figure 3. Characteristics of the liposomes. A: The hydrodynamic diameter, PDI and zeta potential of the liposomes (blank or siRNA-loaded) were measured by DLS. SiRNA entrapment efficiency was measured by RiboGreen assay. **B**: Representative TEM image of siRNA-loaded liposomes. **C**: Hydrodynamic diameter distribution of siRNA-loaded liposomes. **D**: Colloid stability of siRNA-loaded liposomes in PBS.

Supplemental Figure 4. A: CCK-8 assay to check the effect of liposomes on the viability of fibroblasts**. B:** Representative results of Tunel assay in the mice lung sections. **C:** Histological analysis of lung, heart, liver, spleen, kidney, and intestine in mice after induction with liposomes. Representative images for H&E. Images were captured at ×400 magnification. **D:** liver, renal, and cardiac function of mice after induction with liposomes**.** Seven mice were included in each group. The data are represented as the mean ± SEM. ALT: Alanine aminotransferase; AST: Aspartate aminotransferase; BUN: Blood Urea Nitrogen; CR: Creatinine; CK: Creatine Kinase.

Supplemental Figure 5. RT-PCR analysis of *Fibronectin*, *Col1a1*, *α-SMA* and *Srpx2* expression in mice after BLM induction with Scrambled or *Srpx2* siRNA-loaded liposomes. Six mice were included in each study group. The data are represented as the mean ± SEM. *, $p < 0.05$; **, $p < 0.01$; ***, $p < 0.001$.

Supplemental Figure 6. A: Western blot analysis of Arginase 1 (alternative activated macrophages marker) expression in mice after BLM induction with Scrambled or *Srpx2* siRNA-loaded liposomes. **B**: Western blot analysis of Srpx2 expression in macrophages following IL-4 induction. Six mice were included in each study group. The data are represented as the mean ± SEM.

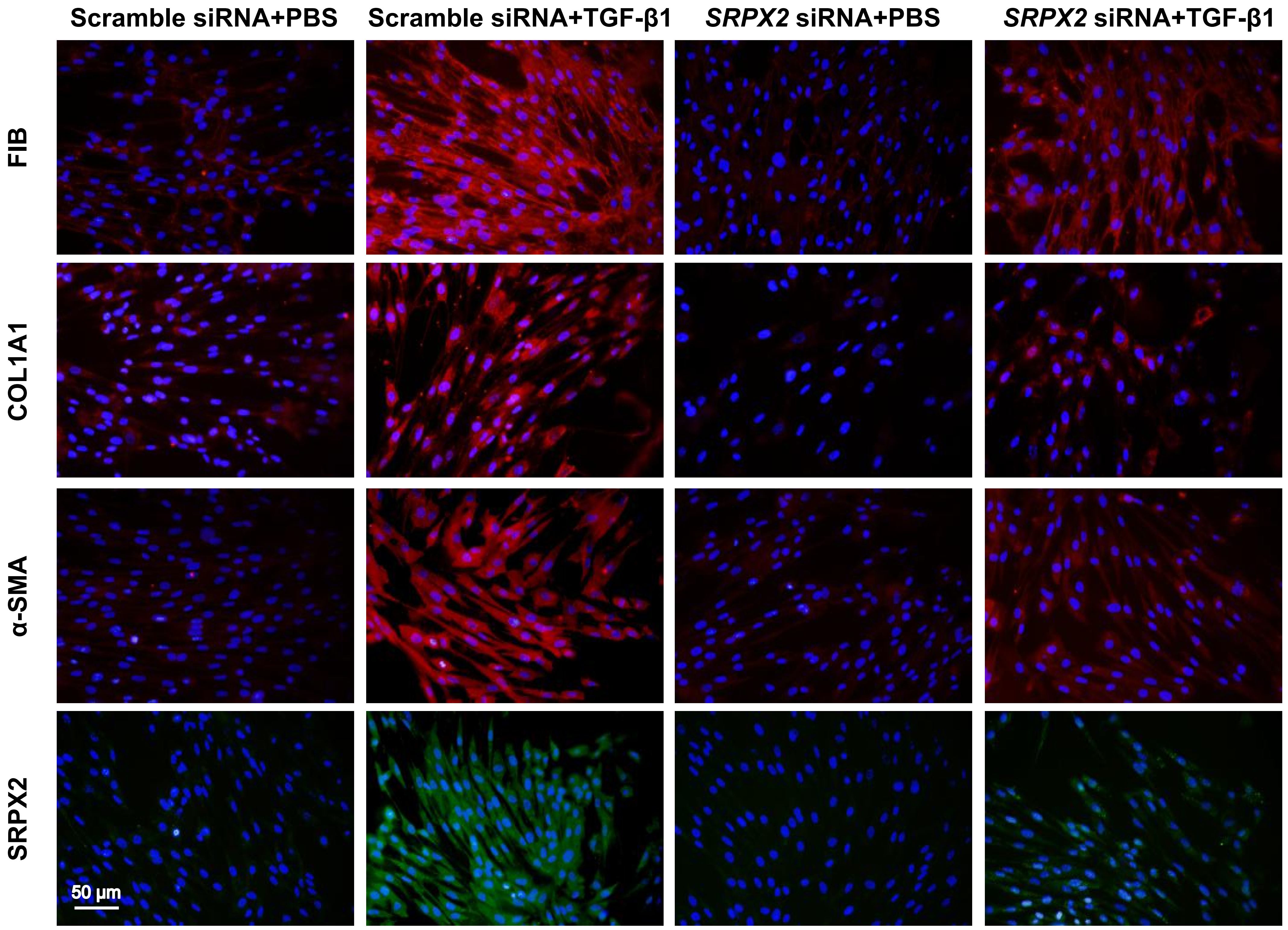
Scramble siRNA+PBS
Scramble siRNA+TGF-β1
SRPX2 siRNA+PBS
SRPX2 siRNA+TGF-β1
FIB
COL1A1
α-SMA
SRPX2
50 μm

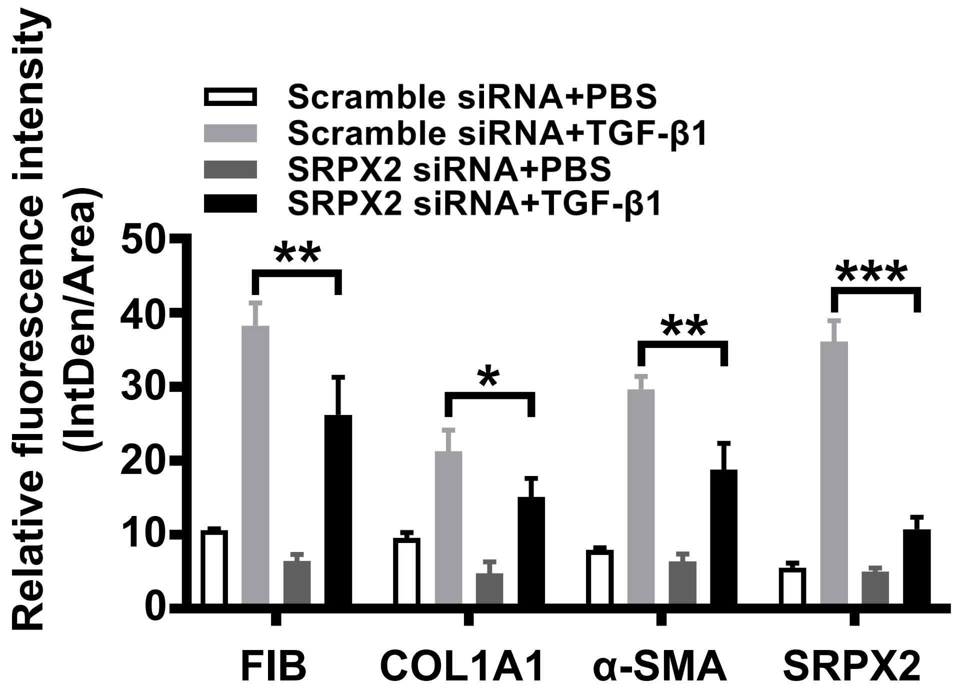
Relative fluorescence intensity
(IntDen/Area)
Scramble siRNA+PBS
Scramble siRNA+TGF-β1
SRPX2 siRNA+PBS
SRPX2 siRNA+TGF-β1
50
40
30
20
10
0
**
*
**
***
FIB
COL1A1
α-SMA
SRPX2

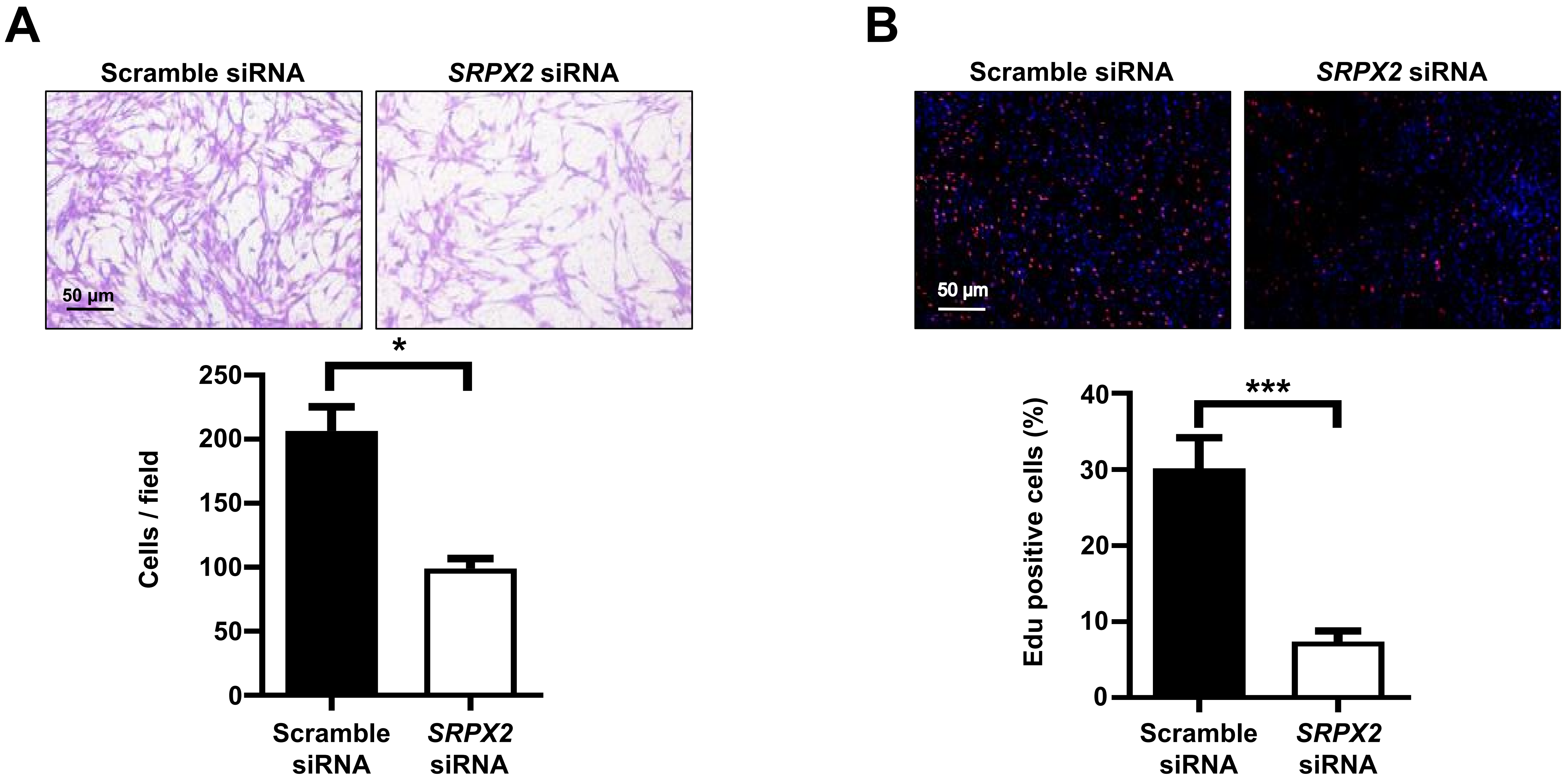
A
Scramble siRNA
SRPX2 siRNA
50 µm
*
250
200
150
100
50
0
Cells / field
Scramble siRNA
SRPX2 siRNA
B
Scramble siRNA
SRPX2 siRNA
50 µm
***
40
30
20
10
0
Edu positive cells (%)
Scramble siRNA
SRPX2 siRNA

A

	Blank liposome	siRNA loaded liposome
Hydrodynamic diameter (nm)	115	102
PDI	0.18	0.07
Zeta-potential (mv)	22.1	2.8
Entrapment efficiency (%)	/	>95

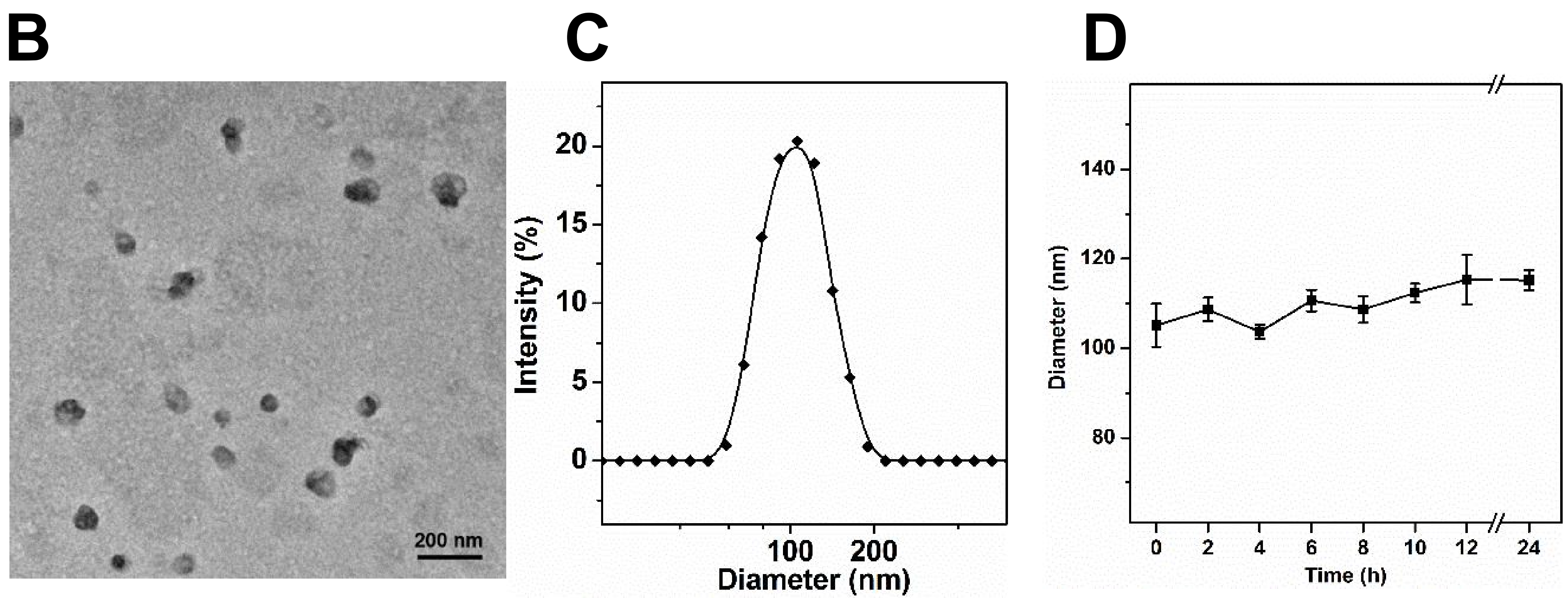

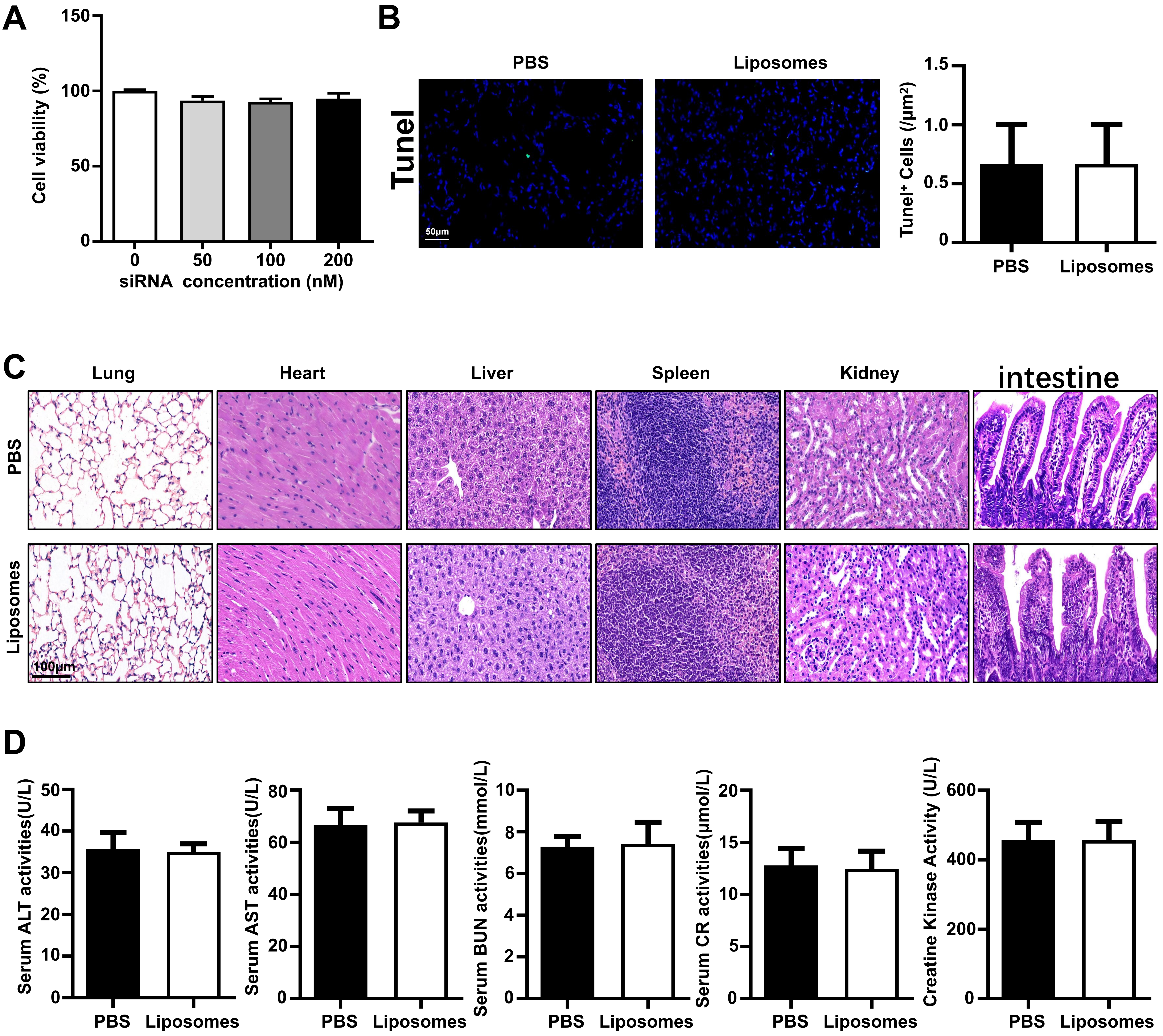
A
150
100
50
0
Cell viability (%)
0
50
100
200
siRNA concentration (nM)
B
PBS
Liposomes
Tunel
50μm
1.5
1.0
0.5
0
Tunel+ Cells (/μm2)
PBS
Liposomes
C
Lung
Heart
Liver
Spleen
Kidney
intestine
PBS
Liposomes
100μm
D
Serum ALT activities(U/L)
50
40
30
20
10
0
PBS
Liposomes
Serum AST activities(U/L)
80
60
40
20
0
PBS
Liposomes
Serum BUN activities(mmol/L)
10
8
6
4
2
0
PBS
Liposomes
Serum CR activities(μmol/L)
20
15
10
5
0
PBS
Liposomes
Creatine Kinase Activity (U/L)
600
400
200
0
PBS
Liposomes

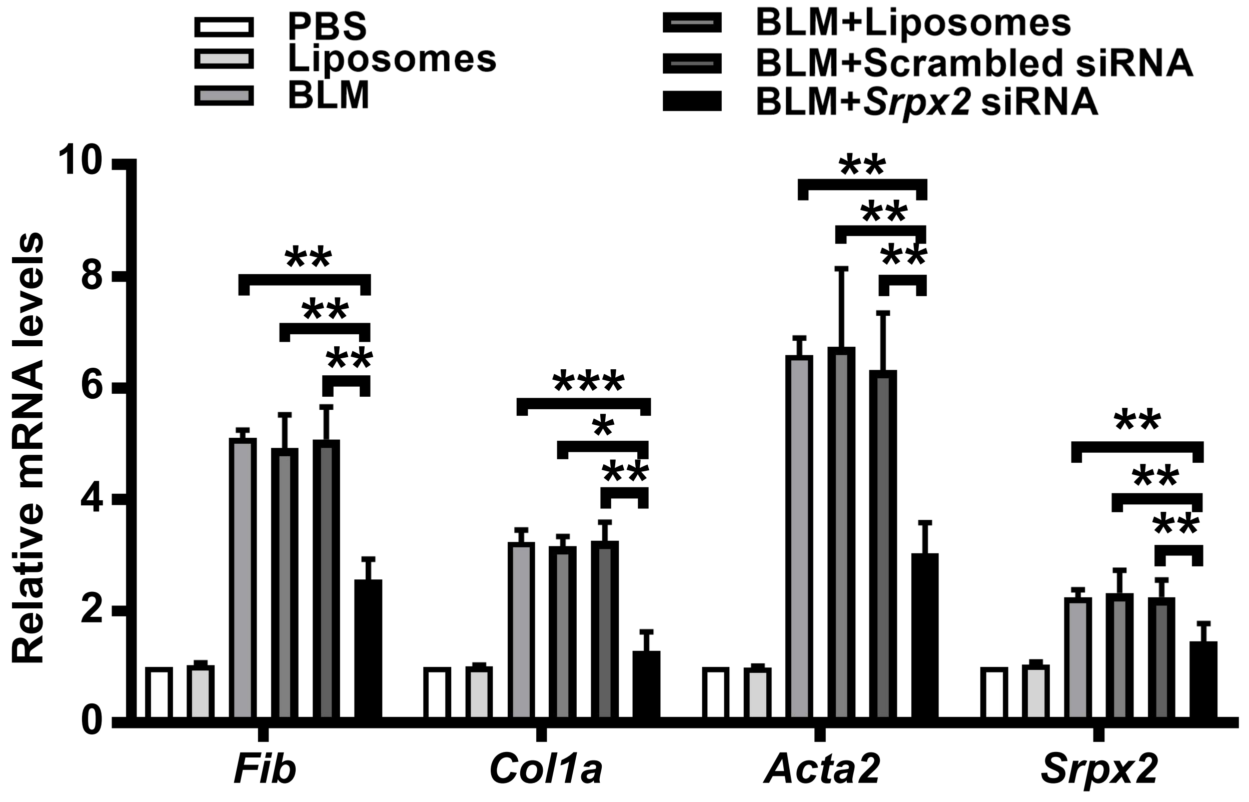

PBS
Liposomes
BLM
BLM+Liposomes
BLM+Scrambled siRNA
BLM+Srpx2 siRNA
Relative mRNA levels
10
8
6
4
2
0
Fib
Col1a
Acta2
Srpx2
**
*
***

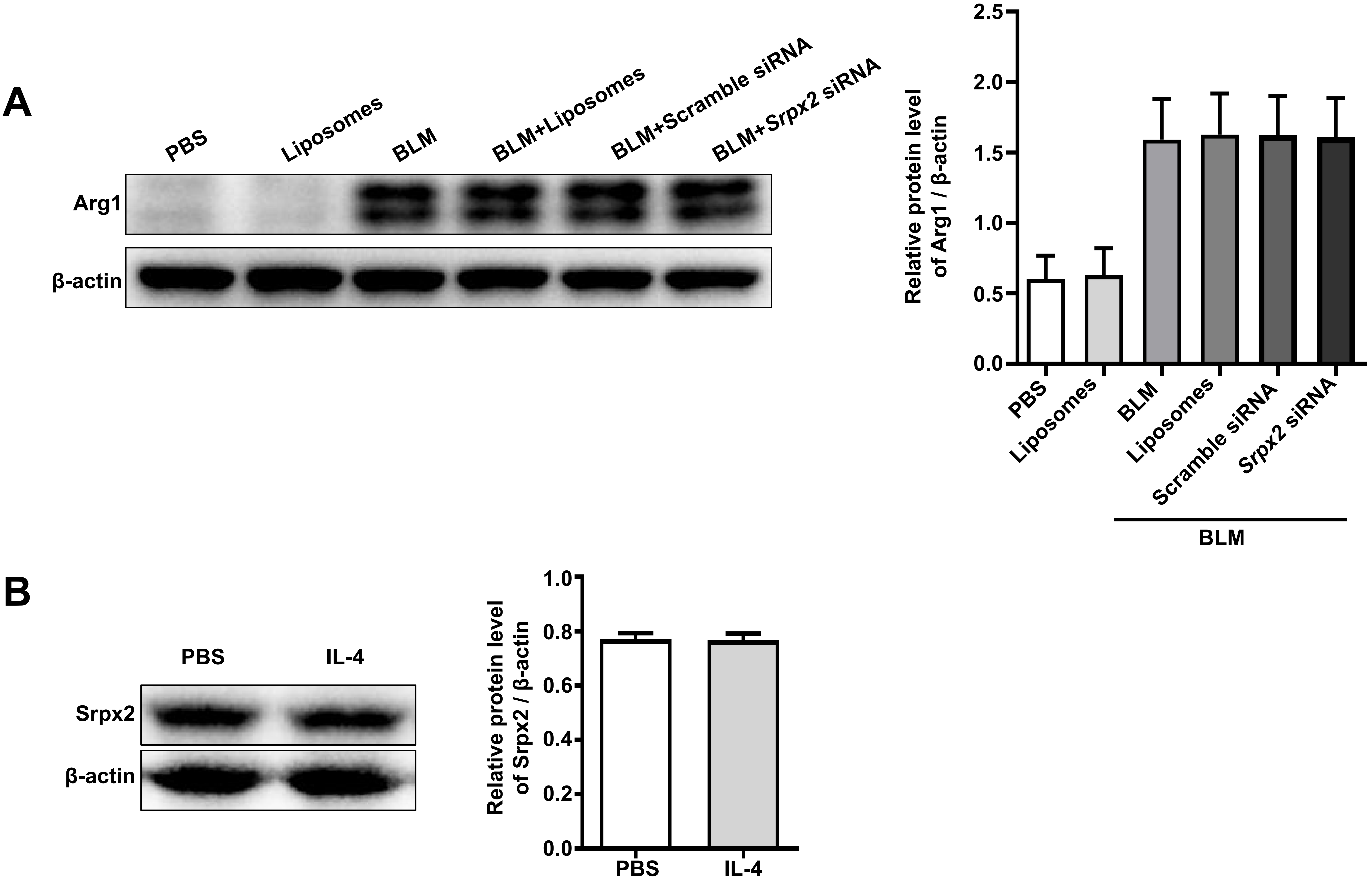

A
PBS
Liposomes
BLM
BLM+Liposomes
BLM+Scramble siRNA
BLM+Srpx2 siRNA
Arg1
β-actin
Relative protein level of Arg1 / β-actin
2.5
2.0
1.5
1.0
0.5
0.0
PBS
Liposomes
BLM
Liposomes
Scramble siRNA
Srpx2 siRNA
BLM
B
PBS
IL-4
Srpx2
β-actin
Relative protein level of Srpx2 / β-actin
1.0
0.8
0.6
0.4
0.2
0.0
PBS
IL-4
